# Promising approach to reducing Malaria transmission by ivermectin: Sporontocidal effect against *Plasmodium vivax* in the South American vectors *Anopheles aquasalis* and *Anopheles darlingi*

**DOI:** 10.1371/journal.pntd.0006221

**Published:** 2018-02-14

**Authors:** Yudi T. Pinilla, Stefanie C. P. Lopes, Vanderson S. Sampaio, Francys S. Andrade, Gisely C. Melo, Alessandra S. Orfanó, Nágila F. C. Secundino, Maria G. V. B. Guerra, Marcus V. G. Lacerda, Kevin C. Kobylinski, Karin S. Escobedo-Vargas, Victor M. López-Sifuentes, Craig A. Stoops, G. Christian Baldeviano, Joel Tarning, Gissella M. Vasquez, Paulo F. P. Pimenta, Wuelton M. Monteiro

**Affiliations:** 1 International Center for Clinical Research in Malaria, Fundação de Medicina Tropical Dr. Heitor Vieira Dourado, Manaus, Brazil; 2 Escola Superior de Ciências da Saúde, Universidade do Estado do Amazonas, Manaus, Brazil; 3 Instituto de Pesquisas Leônidas & Maria Deane, Fundação Oswaldo Cruz, Manaus, Brazil; 4 Centro de Pesquisas René Rachou, Fundação Oswaldo Cruz, Belo Horizonte, Brazil; 5 Department of Entomology, Armed Forces Research Institute of Medical Sciences, Bangkok, Thailand; 6 Department of Entomology, U.S. Naval Medical Research Unit No. 6 (NAMRU-6), Callao, Peru; 7 Mahidol-Oxford Tropical Medicine Research Unit, Faculty of Tropical Medicine, Mahidol University, Bangkok, Thailand; 8 Centre for Tropical Medicine and Global Health, Nuffield Department of Clinical Medicine, University of Oxford, Oxford, United Kingdom; Institut Pasteur, FRANCE

## Abstract

**Background:**

The mosquito resistance to the insecticides threatens malaria control efforts, potentially becoming a major public health issue. Alternative methods like ivermectin (IVM) administration to humans has been suggested as a possible vector control to reduce *Plasmodium* transmission. *Anopheles aquasalis* and *Anopheles darlingi* are competent vectors for *Plasmodium vivax*, and they have been responsible for various malaria outbreaks in the coast of Brazil and the Amazon Region of South America.

**Methods:**

To determine the IVM susceptibility against *P*. *vivax* in *An*. *aquasalis* and *An*. *darlingi*, ivermectin were mixed in *P*. *vivax* infected blood: (**1)** Powdered IVM at four concentrations (0, 5, 10, 20 or 40 ng/mL). (**2**) Plasma (0 hours, 4 hours, 1 day, 5, 10 and 14 days) was collected from healthy volunteers after to administer a single oral dose of IVM (200 μg/kg) (**3**) Mosquitoes infected with *P*. *vivax* and after 4 days was provided with IVM plasma collected 4 hours post-treatment (**4**) *P*. *vivax*-infected patients were treated with various combinations of IVM, chloroquine, and primaquine and plasma or whole blood was collected at 4 hours. Seven days after the infective blood meal, mosquitoes were dissected to evaluate oocyst presence. Additionally, the *ex vivo* effects of IVM against asexual blood-stage *P*. *vivax* was evaluated.

**Results:**

IVM significantly reduced the prevalence of *An*. *aquasalis* that developed oocysts in 10 to 40 ng/mL pIVM concentrations and plasma 4 hours, 1 day and 5 days. In *An*. *darlingi* to 4 hours and 1 day. The *An*. *aquasalis* mortality was expressively increased in pIVM (40ng/mL) and plasma 4 hours, 1, 5 10 and 14 days post-intake drug and in *An*. *darlingi* only to 4 hours and 1 day. The double fed meal with mIVM by the mosquitoes has a considerable impact on the proportion of infected mosquitoes for 7 days post-feeding. The oocyst infection prevalence and intensity were notably reduced when mosquitoes ingested blood from *P*. *vivax* patients that ingested IVM+CQ, PQ+CQ and IVM+PQ+CQ. *P*. *vivax* asexual development was considerably inhibited by mIVM at four-fold dilutions.

**Conclusion:**

In conclusion, whole blood spiked with IVM reduced the infection rate of *P*. *vivax* in *An*. *aquasalis* and *An*. *darlingi*, and increased the mortality of mosquitoes. Plasma from healthy volunteers after IVM administration affect asexual *P*. *vivax* development. These findings support that ivermectin may be used to decrease *P*. *vivax* transmission.

## Author summary

Malaria is one of the most important infectious diseases in the world with hundreds of millions of new cases every year. The disease is caused by parasites of the genus *Plasmodium* where *Plasmodium vivax* represent most of the cases in the Americas. Current strategies to combat malaria transmission are being implemented; however, widespread insecticide resistance in vectors threatens the effectiveness of vector control programs. Ivermectin (IVM) has arisen as a new potential tool to be added to these programs as it has mosquito-lethal and sporontocidal properties making it a promising transmission reduction drug. *Plasmodium vivax* was drawn from patients, mixed with powdered IVM and metabolized IVM in plasma collected from healthy volunteers receiving IVM, and fed to mosquitoes via membrane feeding. Powdered and metabolized IVM interrupt *P*. *vivax* transmission, reducing oocyst infection and intensity rate of two South American malaria vectors *An*. *aquasalis* and *An*. *darlingi*. We also demonstrate the effect of IVM on asexual stages development of *P*. *vivax*, providing evidence that IVM may affect different parasite life cycle stages. Our findings place IVM as a strong candidate for malaria transmission reducing interventions.

## Introduction

The 2016 World Malaria Report (WHO) estimated 212 million cases of malaria worldwide, leading to 429,000 deaths, which illustrates that malaria remains an important public health problem. In the Americas, 389,390 cases and 87 deaths were reported in 2016 with Brazil reporting 24% and Peru 19% of the malaria cases [1]. The majority (92.5%) of the malaria cases occurred in the Amazon sub-region with 69% being *Plasmodium vivax* [1–4]. Despite considerable efforts, the majority of South American countries are still far from achieving vivax malaria elimination. Current strategies to combat malaria transmission in South America include diagnosis and treatment with artemisinin-based combination therapy (ACT) [5–7] and long-lasting insecticidal nets [8], supported by indoor-residual spraying of insecticide IRS [9, 10]. However, widespread insecticide resistance in vectors threatens the effectiveness of LLINs and IRS [1–15].

*Anopheles aquasalis* and *Anopheles darlingi* are known to be susceptible to *P*. *vivax* infection ([16–18]). *Anopheles aquasalis* is considered the primary vector in coastal areas of Central and South America and has been used as a neotropical anopheline vector for laboratory model to evaluate host-parasite interactions [18–20]. *Anopheles darlingi* is the primary vector in the Amazonian region of South America [21, 22].

The reemergence of attention on transmission blocking strategies for *Plasmodium* [23, 24] have raised research efforts in attempt to find vaccines [25–27], drugs [28, 29] or microorganisms [30–32] able to disrupt the life cycle of the parasite in the mosquito vector. In this context, the endectocide ivermectin (IVM) has arisen as a new promising tool to be added to malaria control programs.

Ivermectin is a safe drug with activity against a wide range of internal and external parasites and it is used widely in both veterinary and human medicine [33]. Ivermectin as a single oral dose (150–200 μg/kg) is effective for the treatment or control of *Onchocerca volvulus*, *Wuchereria bancrofti* and *Strongyloides stercoralis*. Ivermectin has been widely distributed to humans via mass drug administration (MDA) campaigns against onchocerciasis and lymphatic filariasis in Africa and Latin America [34–36]. Ivermectin has a secondary effect on ectoparasites that feed on recently treated individuals [37], including activity against *Anopheles* vectors at concentrations present in human blood after standard doses [38, 39]. Consequently, IVM has emerged as a potential tool for malaria control [40, 41]. Ivermectin MDA provides a unique insecticide dissemination route via mosquito ingestion of the compound through a blood meal rather than physical contact as most insecticides are delivered. In *Anopheles* vectors, IVM acts as an agonist of glutamate-gated chloride channels, causing flaccid paralysis and eventual death [42]. Both *in vitro* and *in vivo* studies have shown that a blood meal containing IVM cause a significant reduction in the adult *Anopheles* lifespan, with secondary sub-lethal effects delaying time to re-feed [38, 43], a sporontocidal effect [38, 44, 45], reductions in fecundity [39, 46, 47] and egg hatch rate [39], and even reduced locomotor activity [48].

Field studies of the effect of MDA with IVM on malaria transmission showed that a single dose of 150–200 μg/kg reduced the survivorship of wild *Anopheles gambiae*, with an accompanying reduction in the *Plasmodium falciparum* sporozoite rate [9, 49, 50]. These studies demonstrated that IVM could be a potential additional tool for malaria control.

Several studies have demonstrated that powdered IVM p(IVM) inhibits *Plasmodium* development in the vector, including *P*. *falciparum* in *An*. *gambiae* [44, 45] and more recently *P*. *vivax* in *Anopheles dirus* and *Anopheles minimus* [38] and *An*. *darlingi* [43]. Recent evidence suggests the presence of long-lived IVM metabolites that may have mosquito-lethal activity [38]. Thus, it is critical to determine the sporontocidal effect of IVM and potential metabolites in human plasma/blood after administration of IVM at clinically relevant doses.

Ivermectin has also been shown to inhibit the development of asexual blood stages of *P*. *falciparum in vitro* and *Plasmodium berghei in vivo* [51]. Additionally, it has been reported that IVM has liver stage inhibition in a *P*. *berghei* rodent model [52]. It remains to be determined if these blood and liver stage effects of IVM have a prophylactic personal protective effect for a human.

In this study, for the first time, we evaluated the IVM effect and its possible metabolites against *P*. *vivax* in *An*. *aquasalis* and *An*. *darlingi*. Additionally, we investigated the potential effects of IVM against asexual blood stages of *P*. *vivax*. A better understanding of the effects of IVM and possible metabolites on parasite maturation and transmission to mosquito vectors would contribute to the development of IVM for malaria control strategies.

## Methods

### Ethics statement

The procedures were approved by the Fundação de Medicina Tropical Dr. Heitor Vieira Dourado (FMT-HVD) Ethics Review Board (ERB) (Approval Number: 296.723 CAAE: 14148813.7.0000.0005) and by the U.S. Naval Medical Research Unit No. 6 (NAMRU-6) and Walter Reed Army Institute of Research Institutional Review Boards (NMRCD.2008.0004 and WRAIR#2175). All subjects provided and signed written informed consent.

### *P*. *vivax* malaria patients, healthy volunteer recruitment

This study was conducted at FMT-HVD, a tertiary care center for infectious diseases in Manaus, Amazonas State, Brazil and NAMRU-6 in Iquitos (Loreto), Peru. In order to obtain *P*. *vivax* samples, adult patients (ages ≥18 years) infected with *P*. *vivax* were recruited from the areas surrounding Manaus and Iquitos. *P*. *vivax* infection was determined by light microscopy of Giemsa stained blood samples. In Brazil, *P*. *vivax* infected patients were identified at FMT-HVD, enrolled and venous blood (15 ml) was collected. In Peru, *P*. *vivax* infected patients were identified at Ministry of Health Centers and hospitals in Iquitos, transported to NAMRU-6, enrolled and venous blood (15 ml) was drawn on site for the ivermectin sporogony experiments. The age, gender, history of previous malaria episodes, and place of residence were obtained from the patients and it was verified no signs of severe disease and no previous antimalarial treatment during the preceding 4 weeks. After blood collection, all patients were treated for *P*. *vivax* infection following guidelines of the Brazilian Health Ministry or Peruvian Ministry of Health [53].

In order to obtain metabolized (mIVM) plasma samples, healthy volunteers were recruited in Manaus, Brazil. Inclusion criteria were: adults ≥18 years old, history of at least 3 month-negative malaria, and were confirmed as healthy volunteers by health works.

### Mosquito colonies

*Anopheles aquasalis* were reared at Laboratory of Medical Entomology at FMT-HVD in Manaus, Brazil and *An*. *darlingi* were reared at NAMRU-6 in Iquitos, Peru. These colonies were kept at a constant temperature (24–26°C) and relative humidity (70–80%) on a 12:12 light-dark cycle. Larvae were hatched in room temperature water and fed ground TetraMin fish food for *An*. *aquasalis* and rodent diet for *An*. *darlingi* provided daily. The larvae were allowed to pupate and emerge into adults in an enclosed mesh-covered cage with water and 10% sucrose available [18, 54]. Adult mosquitoes used for experiments were between 3–5 days post-emergence.

### Drugs and treatment regimens

Powdered pIVM and powdered chloroquine (pCQ) reference material were obtained from Sigma Aldrich (St. Louis, MO, USA). pIVM was dissolved in dimethyl sulfoxide (DMSO) to a concentration of 10 mg/ml and pCQ in Roswell Park Memorial Institute (RPMI) 1640 medium (Sigma Aldrich, St. Louis, MO, USA) to a concentration of 1 mg/ml and aliquots were frozen at -20°C. IVM was serially diluted in PBS to achieve experimental concentrations.

All experimental drug regimens were managed at the FMT-HVD in Manaus, Brazil. IVM tablets (Abbot Laboratórios do Brasil, State, Brazil) were administered at a single dose of 200 μg/kg. Vivax patients received chloroquine (CQ) tablets (Farmaguinhos Laboratórios do Brasil, Rio de Janeiro State, Brazil), administered as a daily dose for three days (i.e., 600 mg in the first day and 450 mg in the second and third day). Primaquine (PQ) tablets (Med Pharma, São Paulo State, Brazil) were administered as a daily dose of 30 mg for 7 days following the Brazilian MoH guideline for vivax malaria treatment [53].

To evaluate the effect of mIVM on *P*. *vivax* development, five volunteers, all healthy men from 18 to 50 years, were recruited for the experiments. They received one single dose (200 μg/kg) of IVM. Blood was collected in heparinized tubes at 0 and 4 hours, and 1, 5, 10 and 14 days after drug intake.

For the evaluation of the *in vivo* mIVM effect on *P*. *vivax*, 15 patients with confirmed *P*. *vivax* malaria infection were recruited. The patients were divided into four groups and different treatment regimens were provided: (1) IVM plus CQ, (2) CQ alone, (3) PQ plus CQ and (4) IVM plus PQ plus CQ. Before and after 4 hours of drug intake, blood samples were collected. The patients that receive IVM plus CQ or CQ alone have received the first PQ dose after blood was drawn at 4 hours past CQ intake. All patients were treated with PQ and CQ dosage following the Brazilian Ministry of Health Guidelines.

### Transmission blocking experimental design

**Fig 1:**
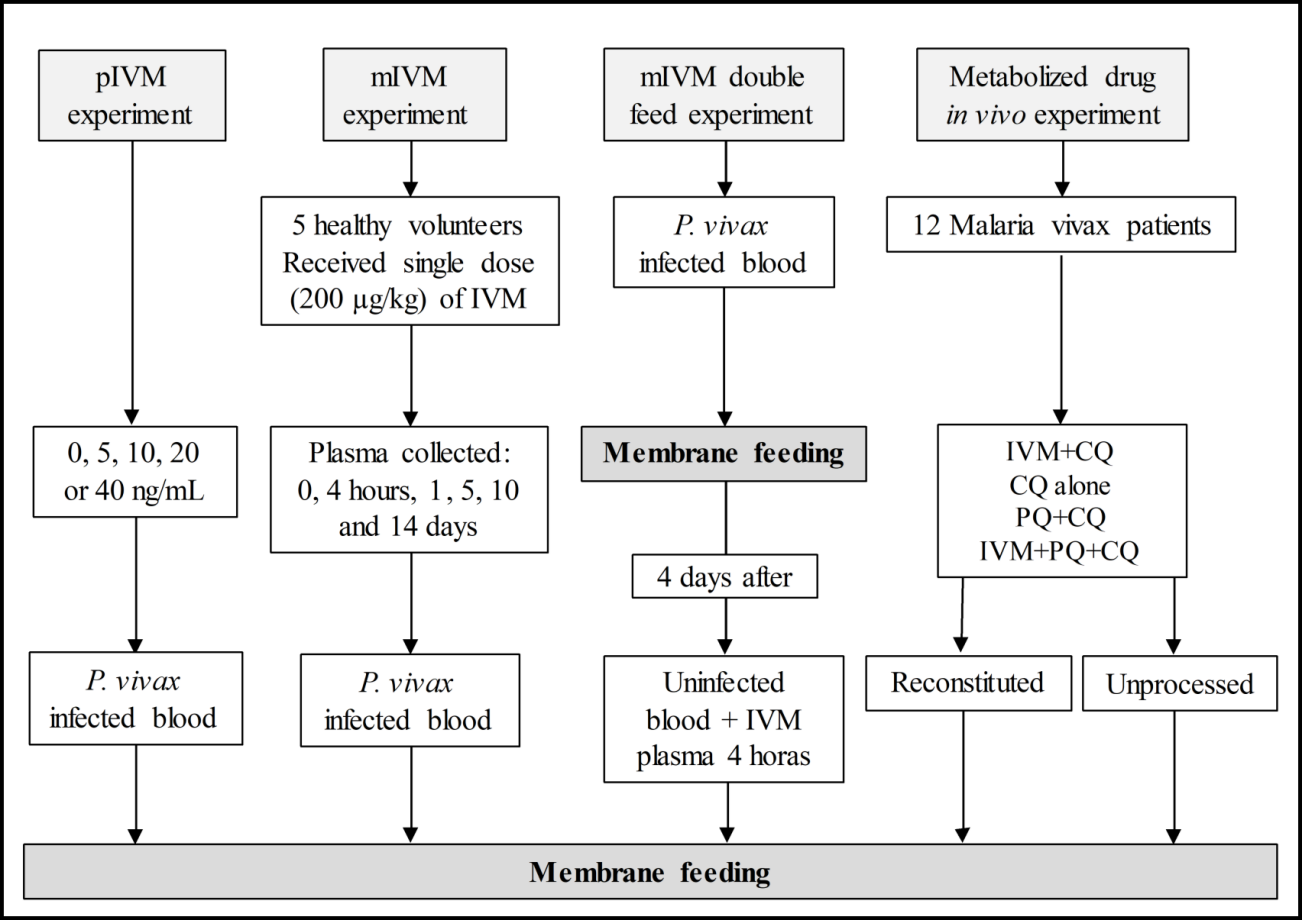
Schematic outline of the experiments of membrane feeding. Four experiments were performed to determine the transmission blocking potential of IVM in either *An*. *aquasalis* or *An*. *darlingi*. Abbreviations: Ivermectin (IVM), powdered ivermectin (pIVM), metabolized ivermectin (mIVM), chloroquine (CQ), primaquine (PQ).

Four experiments were performed to determine the effect of IVM on *P*. *vivax* in either *An*. *aquasalis* or *An*. *darlingi* (Fig 1):

1. Powdered ivermectin experiment (pIVM)—*P*. *vivax* infected blood was maintained at 37°C until centrifuged at 1,500 RPM for 5 minutes at 37°C. The plasma was removed and packed red blood cells (RBCs) were washed with RPMI 1640 medium, repeated twice, and reconstituted to 40% hematocrit with non-immune human AB serum (Sigma Aldrich, MO, USA) and mixed with pIVM to achieve final blood concentrations of 5, 10, 20 or 40 ng/mL and a control (0 ng/ml). These concentrations were chosen based on the human pharmacokinetic curve for IVM, corresponding approximately to the concentration founds 4 hours to 60 hours post ingestion [33, 55]. These experiments were only performed with *An*. *aquasalis* at FMT-HVD.
2. Metabolized ivermectin (mIVM) experiment—*P*. *vivax* infected blood meal was mixed with plasma from the healthy volunteers administered ivermectin. Ivermectin-treated blood was collected at 0 and 4 hours and 1, 5, 10 and 14 days after administration. Ivermectin-treated blood from the healthy volunteers was centrifuged at 1,500 RPM for 5 min and plasma was removed and stored frozen at -20°C. These experiments were performed with *An*. *aquasalis* at FMT-HVD and *An*. *darlingi* at NAMRU-6.
3. Metabolized ivermectin (mIVM) double feed experiment—Mosquitoes were infected with *P*. *vivax* and then four days later were blood fed mIVM collected 0 hours (control) or 4 hours after drug administration. These experiments were performed with *An*. *aquasalis* at FMT-HVD and *An*. *darlingi* at NAMRU-6.
4. Metabolized drug *in vivo* experiment—*P*. *vivax* infected patients were treated with the following drug regimens described above: IVM+CQ, CQ alone, PQ+CQ and IVM+PQ+CQ. To elucidate the sporontocidal effect of IVM, CQ, and PQ after *in vivo* exposure in humans, three different blood meal preparations were made from the same patient: reconstituted, unprocessed, and control. For reconstituted blood preparations, *P*. *vivax* infected blood was collected just before drug administration, held at 37°C for 4 hours and the blood was reconstituted with drug-treated plasma from the same patient collected 4 hours later. The 4th hour drug-treated blood samples were centrifuged at 1,500 RPM for 5 minutes at 37°C. The 0th hour *P*. *vivax* blood was centrifuged, washed with RPMI and reconstituted to a 40% hematocrit with the 4 hours drug-treated plasma and fed to mosquitoes. For unprocessed blood preparations, the blood was collected 4 hours post drug intake and no manipulation of the blood sample was performed prior to feeding to mosquitoes. For control blood preparations, the blood was collected at 0 hours before drug intake and no manipulation of the blood sample was performed prior to feeding to mosquitoes. These experiments were only performed with *An*. *aquasalis* at FMT-HVD.

### *P*. *vivax* infection assessment in mosquitoes

*P*. *vivax* infected blood from patients was prepared as described above. The plasma was removed and packed red blood cells (RBCs) were washed with RPMI 1640 medium, repeated twice, and reconstituted to 40% hematocrit with non-immune human AB serum (experiment 1: pIVM) or with plasma from drug-treated volunteers or patients (experiments 2–4: mIVM, double feed mIVM, and *in vivo* mIVM). Adult female mosquitoes were sugar starved overnight prior to infection via membrane feeding assay (MFA). Blood meals (1ml) prepared as described above were offered to groups of at least 100 mosquitoes for 30 minutes via membrane feeder devices at 37°C as described in detail elsewhere [18, 20]. The fully engorged mosquitoes were separated into different cages and kept until 7 or 14 days post-feeding. Mosquito mortality was monitored on day 7 or 14.

Seven days after *P*. *vivax* blood meal ingestion midguts from all experimentally infected mosquito groups were dissected in PBS under a stereo microscope. The midguts were stained with 0.1% commercial Mercurochrome (Merbromin, Sigma- Aldrich, USA), placed under a cover slip and examined for the presence of oocysts with a compound microscope (Optical Microscopy, Olympus, Germany). Infection prevalence was expressed as percentage of mosquitoes with at least one oocyst. Infection intensity was determined as the arithmetic mean of oocysts counted per dissected midgut.

### Measurement of ivermectin from human plasma

Plasma samples from healthy volunteers and patients were shipped on dry-ice to the Department of Clinical Pharmacology, Mahidol-Oxford Tropical Medicine Research Unit, Bangkok, Thailand for IVM drug concentration measurements. Plasma concentrations of IVM were determined by a newly developed and validated method using solid-phase extraction and liquid chromatography with tandem mass spectrometry (manuscript in preparation). The linear range for quantification was 0.97–384 ng/m. Three replicates of quality control samples at low, middle, and high concentrations were included in the analysis to ensure precision and accuracy. The observed total assay coefficient of variation was <10% in all quality control samples in accordance with US Food and Drug Administration requirements [56].

### *Ex vivo* schizont maturation inhibition assay

The *P*. *vivax* schizont maturation assay was performed as previously described [57] with five *P*. *vivax* samples from Manaus, Brazil. The parasitemia was determined by counting the number of parasites per 200 leukocytes. The plasma and buffy coat were separated from RBCs by centrifugation and packed RBCs were washed with RPMI and this was repeated twice. Leukocytes were removed by passing samples in a cellulose column [58] and afterward, the RBC pellet was suspended in McCoy 5A medium supplemented with 3.2% glucose and 20% human AB serum.

Drug plates were freshly prepared to avoid drug degradation. The pIVM and pCQ stock solutions were subsequently diluted in McCoy’s medium to obtain 9 serial dilutions of the drug (1000–3.9 ng/ml). To determine IC_50_s, 50 μl of pIVM and pCQ solutions were added into 3 wells of 96 well plates, and 9 serial dilutions of each drug were done in triplicate. Three wells were free of drug and served as a control. Additionally, 50 μl of plasma samples from each IVM-treated healthy volunteer 0 and 4 hours after IVM administration were added at 4 different dilutions (1:2, 1:4, 1:8 and 1:16) in triplicate. Then 50 μl of parasite solution was added to each well to achieve a 4% hematocrit. The plates were incubated in a modular incubator chamber containing 5% CO_2_, 5% O_2_, and 90% N_2_ at 37°C. Incubation was stopped when at least 40% of parasites on the ring stage had matured to schizonts in the drug-free control wells.

After incubation, the plates were allowed to stand for 30 minutes in a semi-vertical position. The supernatant was removed, erythrocytes suspended in the remaining fluid, and a thick blood film was made from each well. Thick blood films were stained with Giemsa stain. The number of schizonts containing more than three nuclei per 200 asexual stage parasites was determined in each blood film. Data for analyses prepared as follows: Schizont maturation in relation to control (%) = 100 x (number of schizonts in treated well/number of schizonts in control wells).

### Data analysis

Data were entered in Prism 7.03 (GraphPad, USA) and subsequently analyzed by Stata 11.2 (Data Analysis and Statistical Software, Texas, USA). Mosquito oocyst prevalence and schizont proportions were compared by one-way ANOVA and post-hoc analysis using paired T-test to compare each concentration with respect to the control. Mosquito oocyst intensity was compared by Kruskal-Wallis test and post-hoc analysis using Kolmogorov-Smirnov to compare each concentration with respect to the control. P-values <0.05 were considered statistically significant. The *P*. *vivax* mosquito-stage oocyst inhibition in *An*. *Aquasalis* and *An*. *darlingi* after ingestion of whole blood spiked with IVM or whole blood equivalent from volunteers administered ivermectin were analyzed using GraphPad Prism v7.02. Normalized oocyst inhibition versus human volunteer plasma concentrations of IVM was analyzed using a nonlinear dose-response analysis with a variable slope. The maximum inhibition was fixed to 100%, and the minimum inhibition (zero drug concentration) and the drug concentration producing 50% of maximum effect (IC_50_) was estimated. The *P*. *vivax* asexual blood-stage inhibitory concentrations (i.e. IC_50,_ IC_90_ and IC_99_) for pIVM and pCQ were determined using the free software ICE estimator available online at http://www.antimalarial-icestimator.net/.

## Results

### Plasma ivermectin results

Median peak plasma concentrations (i.e., plasma samples collected 4 hours post-dose) was 79.8 (50.6–112) ng/mL after a single oral dose of 200 μg/kg of IVM. Only two out of five volunteers had detectable IVM plasma concentrations 10 days post-dose (i.e., 2.64 and 2.32 ng/mL), while only one volunteer had a detectable IVM plasma concentration on day 14 after dose (i.e., 1.87 ng/mL) (S1 Table).

### Powdered Ivermectin (pIVM) inhibits *P*. *vivax* development in *An*. *aquasalis*

**Fig 2:**
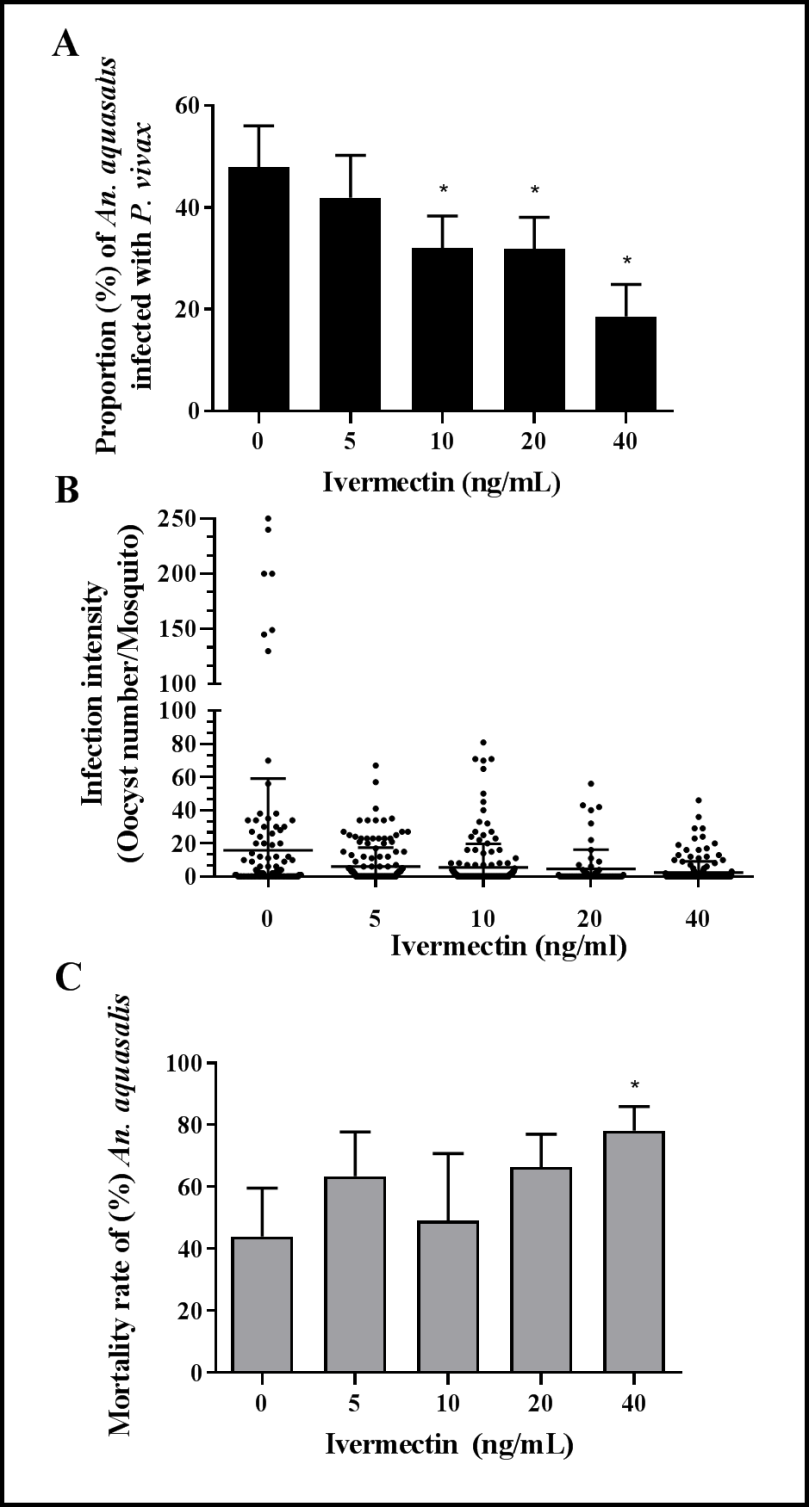
Ivermectin powdered affects *Plasmodium vivax* development in *Anopheles aquasalis*. (A) The proportion of *P*. *vivax* infected mosquitoes is presented as a mean and standard deviation. (B) The infection intensity is presented as the number of oocysts per a single midgut (black dots) and the black lines represent the mean and standard deviation. (C) Mosquito mortality rate to 7 days post-feeding is presented as mean and standard deviation. Data from 5 independent experiments are presented in A-C. Asterisks (*) represent significant difference (P < 0.05) in relation to the control.

Different concentrations of pIVM were evaluated (5, 10, 20 and 40 ng/mL) on the *P*. *vivax* infection prevalence, oocyst intensity, and *An*. *aquasalis* mortality (Fig 2A–2C, S1 Dataset). There were significant differences among pIVM treatment groups for infection prevalence [F (3.23) = 11.58, p = 0.001] compared to the control (0ng/ml) group. *P*. *vivax* infection prevalence was significantly reduced in mosquitoes that ingested pIVM at 10 ng/ml by 33.2% [32.03% (SD = 5.83%), p = 0.0051, reps = 7, n = 171], 20 ng/ml by 33.7% [31.81% (SD = 5.74%), p = 0.019, reps = 7, n = 68] and 40 ng/ml by 61.3% [18.60% (SD = 5.81%), p<0.0001, reps = 7, n = 178) concentrations but not the 5ng/ml by 12.6% [41.93% (SD = 7.67%), p = 0.141, reps = 7, n = 160) concentration (Fig 2A). Also, the infection intensity (i.e. number of oocysts per mosquito) was reduced in the groups of mosquitoes that fed on infective blood meals containing 10ng/mL by 62.6% [5.89 (SD = 5.87), p = 0.0079, reps = 7, n = 171), 20ng/mL by 72.6% [4.32 (SD = 2.71), p = 0.0298, reps = 7, n = 68] and 40ng/mL by 86.1% [2.20 (SD = 0.97), p<0.0001, reps = 7, n = 178) but not the 5ng/ml by 56.6% [6.84 (SD = 3.20), p = 0.99, reps = 7, n = 160) concentration of pIVM in comparison to the control group (Fig 2B) [15.73 (SD = 12.97), reps = 7, n = 134]. There were significant differences among pIVM treatment groups for mosquito mortality at 7-day [F (3.23) = 2.99, p = 0.052] compared to the control (0ng/ml) group but the mortality rate was higher only in the 40 ng/ml group [78.11% (SD = 7.17%), p = 0.0007] but not 20 ng/ml group [66.34% (SD = 9.72%), p = 0.056], 10 ng/ml group [49.09% (SD = 20.03%), p = 0.646] or 5ng/ml group [63.38% (SD = 13.26%), p = 0.080] compared to the control group [43.93% (SD = 14.46%)] (Fig 2C).

### Metabolized ivermectin (mIVM) inhibits *P*. *vivax* development in *An*. *aquasalis* and *An*. *darlingi*

**Fig 3:**
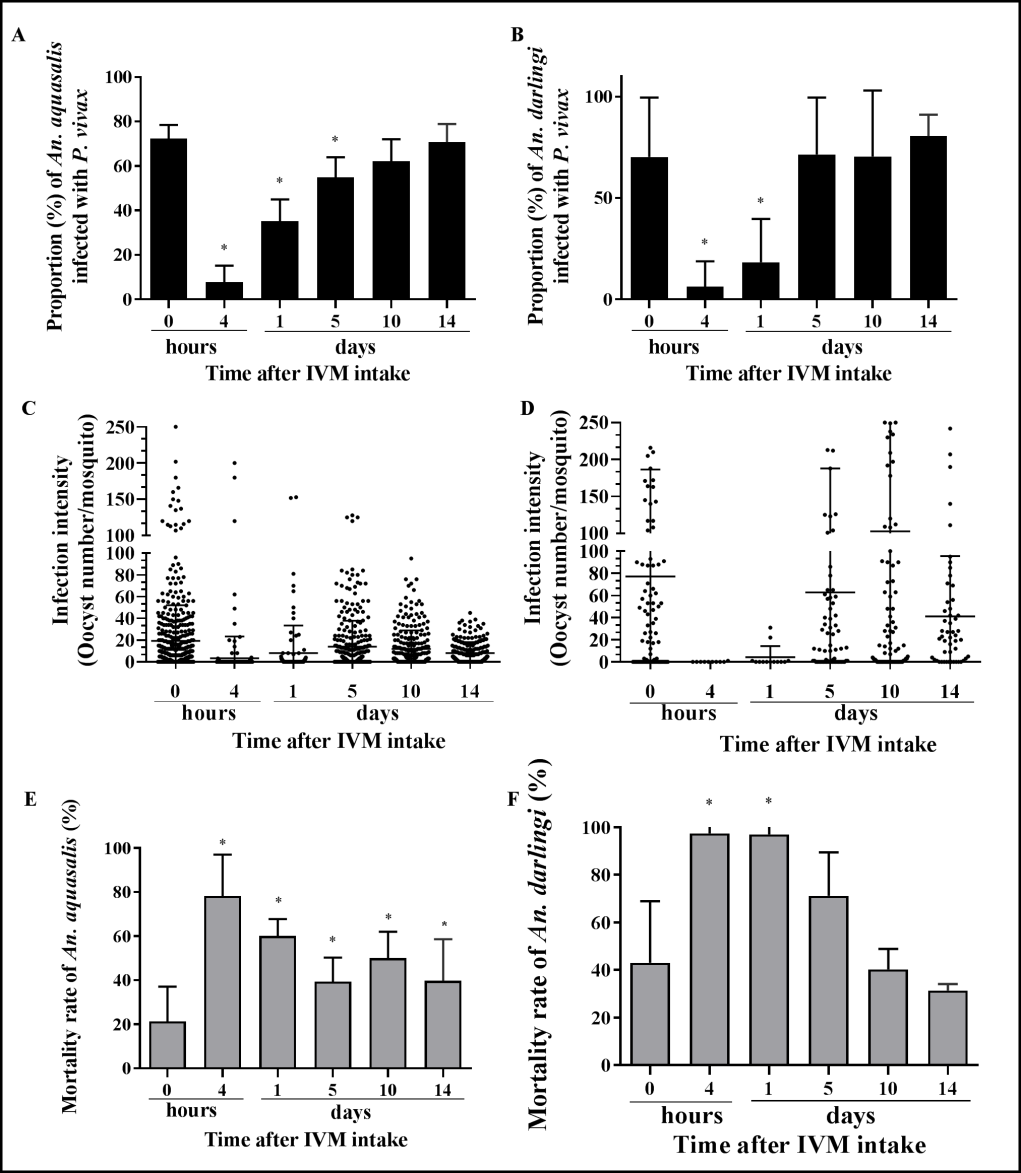
Ivermectin metabolized affects *Plasmodium vivax* development in *Anopheles aquasalis* and *Anopheles darlingi*. *An*. *aquasalis* (A,C,E) and *An*. *darlingi* (B,D,F) mosquitoes were fed *P*. *vivax* with mIVM collected at various time points (0 and 4 hours; 1, 5, 10 and 14 days). (A) and (B) Proportion of *P*. *vivax* infected mosquitoes are presented as mean and standard deviation. (C) and (D) The intensity of infection is presented as the number of oocysts per a single midgut (black dots) and the black lines represent the median and standard deviation. (E) and (F) The mosquito mortality rate is presented as mean and standard deviation. Data from 5 independent experiments are presented. Asterisks (*) represent significant difference (P < 0.05) in relation to the control.

The ingestion of mIVM presented in the infective blood meals affected the infection rate and the infection intensity and the mortality of the vectors *An*. *aquasalis* and *An*. *darlingi* as seen in the Fig 3A–3F. There were significant differences for among mIVM treatment groups for infection prevalence [F (3.31) = 119.02, p<0.001] in *An*. *aquasalis* and [F (2.9) = 9.39, p = 0.0063] in *An*. *darlingi* compared to the control (0 hour) group. The oocyst infection rates were reduced in the groups of *An*. *aquasalis* fed volunteer mIVM plasma collected at 4 hours (48.0 ng/ml) by 89.2% [7.82% (SD = 7.04%), p<0.0001, reps = 10, n = 232), 1 day (5.55 ng/ml) by 51.5% [35.11% (SD = 8.96%), p = 0.0038, reps = 6, n = 102], and 5 days (1.58 ng/ml) by 24.2% [54.88% (SD = 8.57%), p = 0.0009, reps = 9, n = 300) but not 10 days by 14.3% [62.10% (SD = 9.46%), p = 0.063, reps = 9, n = 258) or 14 days by 2.3% [70.75% (SD = 7.57%), p = 0.073, reps = 9, n = 214) (Fig 3A) compared to the mIVM control [72.38% (SD = 6.05%), reps = 10, n = 448]. The oocyst infection rates were reduced in the groups of *An*. *darlingi* fed mIVM plasma collected at 4 hours (43.24 ng/ml) by 91.1% [6.25% (SD = 10.8%), p = 0.019, reps = 4, n = 8] and 1 day (5.69 ng/ml) by 73.9% [18.33% (SD = 18.48%), p = 0.014, reps = 4, n = 11], but increased slightly but not significantly on 5 days (1.35 ng/ml) by 1.95% [71.47% (SD = 22.88%), p = 0.156, reps = 4, n = 67], 10 days (2.48 ng/mL) by 0.48% [70.44% (SD = 28.17%), p = 0.098, reps = 4, n = 97], or 14 days (1.87 ng/mL) by 14.89% [80.54% (SD = 7.5%), p = 0.350, reps = 4, n = 51] compared with the control [70.1% (SD = 25.4%), reps = 4, n = 97] (Fig 3B). The mIVM concentrations imbibed by the mosquitoes were calculated by taking pharmacokinetic values and multiplying by 60% to account for the 40% hematocrit in each blood meal, several volunteers had no detectable ivermectin at days 10 and 14 so no concentrations ingested by mosquitoes could be estimated. We did not find significant differences for infection prevalence among both species in each treatment group: Control (p = 0.9178), 4 horas (p = 0.527), 1 day (0.287), 5 days (0.2883), 10 days (p = 0.734) and 14 days (p = 0.513). However, the mean oocyst intensity for *An*. *darlingi* (Fig 3D) is substantially higher than *An*. *aquasalis* (Fig 3C).

On the other hand, the oocyst infection intensities were reduced in all the groups: plasma 4 hours (43.24 ng/ml) by 96.1% [0.78 (SD = 2.03), p<0.0001, reps = 6, n = 232], 1 day (5.69 ng/ml) by 56.8% [8.52 (SD = 10.09), p<0.0001, reps = 6, n = 102], 5 days (1.35 ng/ml) by 29.4% [13.91 (SD = 11.39), p<0.0001, reps = 6, n = 300], 10 days by 41.3% [11.57 (SD = 5.50), p = 0.008, reps = 6, n = 258] and 14 days by 58.2% [8.24 (SD = 2.55), p = 0.007, reps = 6, n = 214] after the volunteer pIVM intakes compared with the control [19.70 (SD = 13.09), reps = 6, n = 448] in *An*. *aquasalis* (Fig 3C) and in plasma 4 hours (43.24 ng/ml) by 99.9% [0.06 (SD = 0.10), p = 0.003, reps = 4, n = 8], 1 day (5.69 ng/ml) by 97.57% [2.31 (SD = 3.77), p = 0.008, reps = 4, n = 11] and 5 days (1.35 ng/ml) by 56.8% [76.49 (SD = 80.96), p = 0.011, reps = 6, n = 67], but not 10 days by 56.8% [99.77 (SD = 125.9), p = 0.593, reps = 6, n = 97] and 14 days by 56.8% [37.80 (SD = 5.1), p = 0.141, reps = 6, n = 51] after the volunteer pIVM intakes compared with the control [94.8, (SD = 90.18), reps = 4, n = 97] in *An*. *darlingi* (Fig 3D). It is important to highlight that *An*. *darlingi* showed much less oocyst number on 4 hours and 1 day than *An*. *aquasalis*.

At day 7 post blood meal, there were significant differences in the mortality rate among mIVM treatment groups in *An*. *aquasalis* [F (5.46) = 16.45, p<0.0001] and mIVM treatment groups in *An*. *darlingi* [F (2.9) = 16.92, p<0.0001] compared to the control groups (plasma 0 hours). The mosquito mortality was significant higher 4 hours [78.33% (SD = 17.73%), p = 0.0006], 1 day [60.15% (SD = 6.95%), p = 0.0017], 5 days [36.84% (SD = 11.99%), p = 0.046], 10 days [50.01% (SD = 11.44%), p = 0.014] and 14 days [39.83% (SD = 17.58%), p = 0.043] (Fig 3E) than the control group [21.29% (SD = 15.80%)] in *An*. *aquasalis*. However, at day 7 post blood meal only the *An*. *darlingi* groups feds on infective blood meals containing plasma collected 4 hours [97.43% (SD = 2.56%), p = 0.027] and 1 day [97.07% (SD = 3.23%), p = 0.025] had significantly increased mortality rates but not when fed plasma from 5 days [71.23% (SD = 14.91%), p = 0.144], 10 days [40.25% (SD = 7.47%), p = 0.855] or 14 days [31.4% (SD = 1.9%), p = 0.873] compared with the control [43% (SD = 22.45%] (Fig 3F).

At 14 days post blood meal, after mIVM plasma from 4 hours, 1 and 5 days intake all the *An*. *darlingi* had died, therefore sporozoite prevalence at these time points could not be analyzed. Also, there were no significant differences on the mortality rate with respect to control [36.83% (SD = 20.06%)] in the groups that were blood fed mIVM of 10 days [57.66% (SD = 15.21%), p = 0.265] and 14 days [48.65% (SD = 21.67%), p = 0.200]. *An*. *darlingi* sporozoite prevalence at day 14 post blood meal was not reduced with respect to control [90.27% (SD = 13.39%)] in the groups that were blood fed mIVM from 10 days [90.38% (SD = 10.88%), p = 0.992] or 14 days [82.14% (SD = 1.68%), p = 0.824].

**Fig 4:**
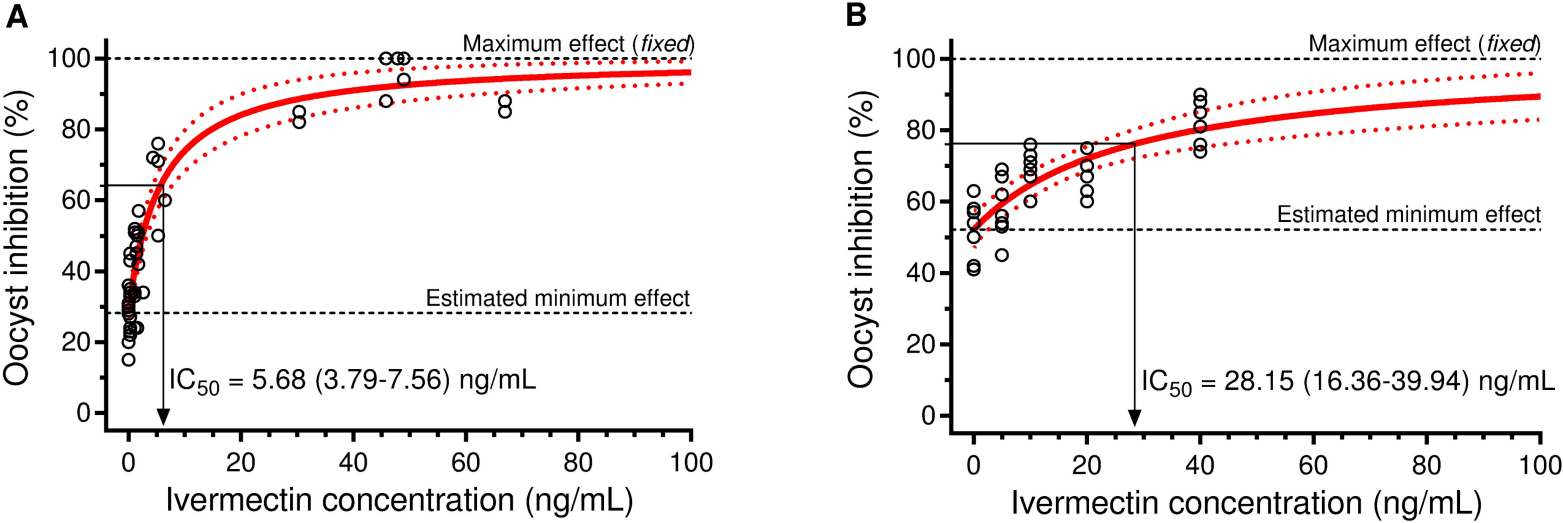
Activity of IVM on *P*. *vivax* infections in *An*. *aquasalis*. *P*. *vivax* oocyst inhibition in *An*. *aquasalis* when ingesting plasma from human volunteers receiving standard single oral dosing of IVM (A) or whole blood spiked with IVM (B).

Interestingly, the mIVM (IC_50_ = 5.68 [3.79–7.56] ng/ml [95%CI]) of mosquito-stage *P*. *vivax* in *An*. *aquasalis* appears to be much lower compared to pIVM (IC_50_ = 28.15 [16.36–39.94] ng/ml) (Fig 4A and 4B). This demonstrates that mIVM has a more potent sporontocidal effect against *P*. *vivax* compared to ivermectin compound.

### mIVM impairs *P*. *vivax* development when ingested 4 days post parasites in *An*. *aquasalis and An*. *darlinigi*

**Fig 5:**
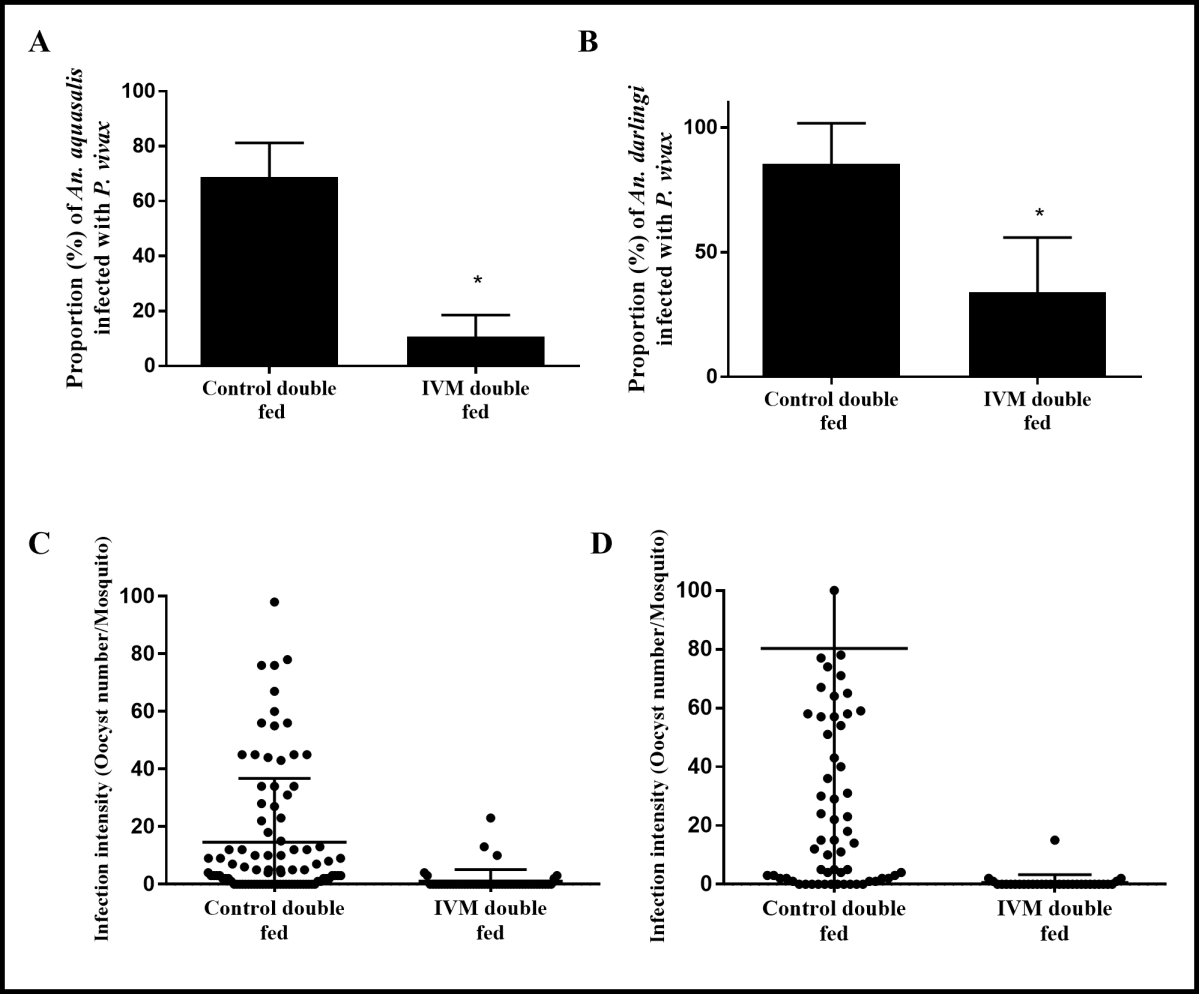
Ivermectin impairs *Plasmodium vivax* development in *Anopheles aquasalis* and *Anopheles darlingi* when administered in a double feed 4 days after infection. *An*. *aquasalis* and *An*. *darlingi* female mosquitos were fed *P*. *vivax* blood samples. Four days after infection, two groups of mosquitos received a second uninfected blood meal with mIVM plasma from control (0 hours) or 4 hours after IVM intake. (A) The proportion of *P*. *vivax* infected *An*. *aquasalis* and (B) *An*. *darlingi* are presented as the mean and standard deviation. (C and D) The intensity of infection is presented as the number of oocysts per midgut (dots) and the black lines represent the median and standard error. Data from 5 independent experiments are presented. Asterisks (*) represent significant difference (P < 0.05) in relation to control.

*Anopheles darlingi* and *An*. *aquasalis* had significantly reduced oocyst infection prevalence and intensity when ingested a second mIVM (4 hours) non-infective bloodmeal 4 days post *P*. *vivax* infection compared to the control. *An*. *aquasalis* oocyst prevalence was reduced by 84.5% [10.72% (SD = 6.79%), p = 0.047, reps = 4, n = 50] compared to the control [68.83% (SD = 10.69%), reps = 4, n = 94] (Fig 5A) and *An*. *darlingi* by 60.3% [33.98% (SD = 17.8%), p<0.0001, reps = 3, n = 31] compared to the control [85.49% (SD = 13.2%), reps = 3, n = 73] (Fig 5B). Oocyst intensity in *An*. *aquasalis* was reduced by 93.6% [0.94 (SD = 0.67), p<0.0001, reps = 4, n = 50] compared to the control [14.65 (SD = 7.71), reps = 4, n = 94] (Fig 5C) and in *An*. *darlingi* by 97% [0.73 (SD = 0.2), p<0.0001, reps = 3, n = 31] compared to the control [24.0 (SD = 53.3), reps = 3, n = 73] (Fig 5D).

### mIVM and metabolized malaria compounds inhibit *P*. *vivax* development in *An*. *aquasalis*

**Fig 6:**
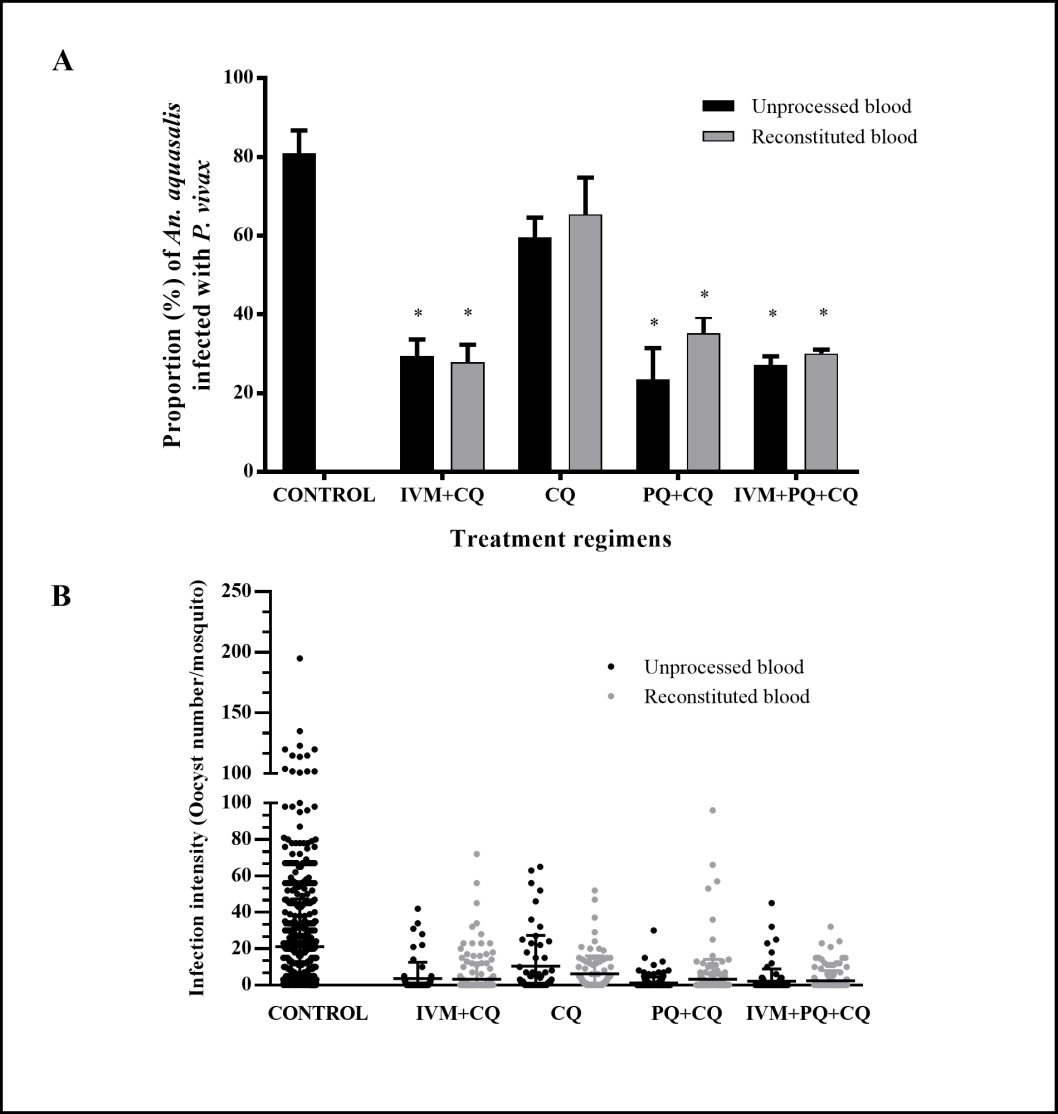
Plasmatic ivermectin and malaria drugs have a direct effect on *Plasmodium vivax* infection in *Anopheles aquasalis*. *An*. *aquasalis* female mosquitos were fed with unprocessed blood samples from *P*. *vivax* patients at time 0 (control) or 4 hours after different treatment regimens intake or with erythrocytes from *P*. *vivax* patients’ blood samples (before treatment) reconstituted to a hematocrit of 40% with the respective plasma after 4 hours of different treatment regimens intake. (A) The proportion of *P*. *vivax* infected mosquitos are presented as mean and standard deviation. Black bars represent the unprocessed blood and the grey bars represent the reconstitute samples. (B) The intensity of infection is presented as the number of oocysts per a single mosquito midgut (dots), black dots represent the unprocessed blood and the grey dots represent the reconstituted blood samples, and the black lines represent the median and standard error blood. Asterisks (*) represent significant difference (P < 0.05) in relation to the control.

There were significant reductions in oocyst infection prevalence and intensity compared to control when *An*. *aquasalis* ingested the drug-treated infected blood meals with unprocessed [F (3.17) = 159.90, p<0.0001] and reconstituted [F (3.17) = 164.82, p<0.0001] blood. For unprocessed blood, IVM+CQ by 63.7% [29.44% (SD = 4.19%), p = 0.029, reps = 3, n = 64]; PQ+CQ by 71.1% [23.44% (SD = 7.95%), p = 0.02, reps = 3, n = 161] and IVM+PQ+CQ by 66.5% [27.15% (SD = 2.20%), p = 0.046, reps = 3, n = 99] but not CQ alone by 26.5% [59.49% (SD = 10.9%), p = 0.072, reps = 3, n = 58] compared with the control [80.91% (SD = 4.12%), reps = 3, n = 615], (Fig 6A). Similar to observed in mosquitos fed with unprocessed blood, the infection rate was significantly reduced on mosquitos fed with *P*. *vivax* blood meal reconstituted with plasma from patients that undertook IVM+CQ by 65.6% [27.86% (SD = 4.44%), p = 0.006, reps = 3, n = 205], PQ+CQ 56.55% [35.21% (SD = 4.17%), p = 0.004, reps = 3, n = 180] and IVM+PQ+CQ 62.9% [30.02% (SD = 1.00%), p = 0.042, reps = 3, n = 140] but not CQ alone by 19.2% [65.40% (SD = 7.63%), p = 0.077, reps = 3, n = 91] in comparison to control [80.91% (SD = 4.12%), reps = 3, n = 615] (Fig 6A). There were no significant differences in oocyst prevalence between the unprocessed and reconstituted treatment regimens [IVM+CQ p = 0.246], [CQ p = 0.784], [PQ+CQ p = 0.120] and [IVM+PQ+CQ p = 0.128]. The infection intensity (oocysts number) was significantly reduced in mosquitos fed with unprocessed: IVM+CQ by 90.1% [1.99 (SD = 2.01), p<0.001, reps = 3, n = 64]; PQ+CQ by 90.8% [1.86 (SD = 1.77), p<0.001, reps = 3, n = 161], IVM+PQ+CQ by 89.1% [2.19 (SD = 0.96), p<0.001, reps = 3, n = 99] and CQ alone by 56.5% [8.71 (SD = 8.92), p<0.001, reps = 3, n = 58] compared with the control [20.02 (SD = 4.05), reps = 3, n = 615] (Fig 6B) and reconstituted with plasma from patients that undertook IVM+CQ by 84.1% [3.19 (SD = 1.43), p<0.001, reps = 3, n = 205], PQ+CQ 71.6% [5.69 (SD = 5.14), p<0.001, reps = 3, n = 180], IVM+PQ+CQ by 87.6% [2.50 (SD = 0.64), p<0.001, reps = 3, n = 140] and [20.02 (SD = 4.05), reps = 3, n = 615]. There were no significant differences in oocyst prevalence between the unprocessed and reconstituted treatment regimens [IVM+CQ p = 0.246], [CQ p = 0.784], [PQ+CQ p = 0.120] and [IVM+PQ+CQ p = 0.128].

### mIVM impairs asexual *P*. *vivax* maturation

**Fig 7:**
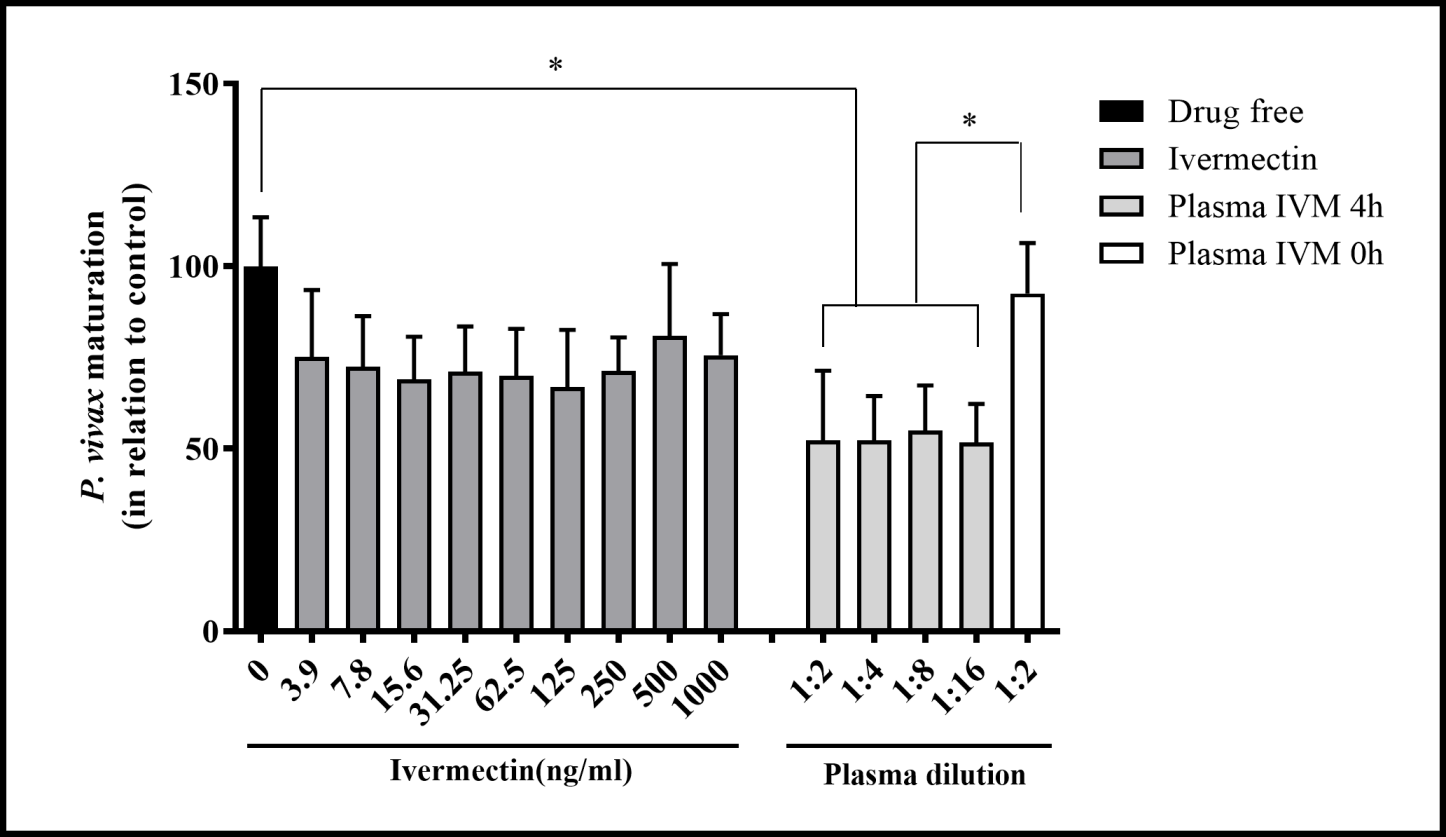
Metabolized ivermectin impairs asexual *P*. *vivax* maturation. Five *P*. *vivax* samples containing more than 80% of parasites on ring stage were evaluated. Nine different concentrations of pIVM were added to *P*. *vivax* culture. Moreover, plasma from healthy volunteers collected before (0 hours) and after 4 hours of IVM intake was added in 4 different serial dilutions (1:2) with complete medium. A drug free condition was used as the control. The number of schizonts in 200 asexual parasites was evaluated for each condition and the maturation in relation to control were determined. Asterisks (*) represents significant differences (P < 0.05) in relation to control by Kruskal-Wallis test.

A total of 5 malaria vivax patients were recruited with high parasitemia (138–245 parasites/200 leucocytes), with at least 80% of parasites in the ring stage. We tested five isolates of *P*. *vivax* for pIVM drug sensitivity. The pIVM had some activity against two of five *P*. *vivax* isolates tested (Table 1). As expected in our site, chloroquine was fully effective as the blood schizonticidal treatment with pCQ IC_50_ ranging from 1.96 ng/mL to 7.53ng/mL. Interestingly, when mIVM was added to *P*. *vivax* culture in different dilutions, a significant reduction in parasite maturation was observed compared to drug free control 1:2 (34.24 ng/ml) [52.31% (SD = 18.06%), p = 0,011, reps = 5]; 1:4 (17.12 ng/ml) [52.34% (SD = 11.49%), p = 0.0002, reps = 5]; 1:8 (8.56 ng/ml) [54.93% (SD = 11.78%), p = 0.0013, reps = 5] and 1:16 (4.28 ng/ml) [51.77% (SD = 9.92%), p = 0.0001, reps = 5] (Fig 7).

**Table 1:** *Ex vivo* activity of ivermectin and chloroquine against *P*. *vivax* isolates from Manaus- AM, Brazil.

Isolate	pIVM (ng/ml)			pCQ (ng/ml)		
	IC_50_	IC_90_	IC_99_	IC_50_	IC_90_	IC_99_
1	14.83	18.48	23.49	7.53	464.13	41687.71
2	NI	NI	NI	5.60	85.92	1693.20
3	3.61	4.5	5.71	1.98	2.47	3.13
4	NI	NI	NI	1.85	2.31	2.93
5	NI	NI	NI	1.96	2.43	3.10

Expressed as IC_50_(ng/ml). NI: no inhibition. pIVM: powdered ivermectin, pCQ: powdered chloroquine

## Discussion

Effective malaria transmission-blocking tools are an integral element in malaria eradication campaigns. MDA of IVM disrupted malaria parasite transmission in West Africa [9, 49] by killing the vector *An*. *gambiae* [9, 50] which shifts the population age structure, thereby reducing the sporozoite rate. Additional effects of ivermectin which likely further reduce transmission include inhibiting sporogony in the vector as demonstrated with *P*. *falciparum* in *An*. *gambiae* [45] and *P*. *vivax* in *An*. *dirus*, *An*. *minimus* [37], and *An*. *darlingi* [43]. Previous findings from our group also showed effects of pIVM and mIVM on survivorship, fecundity and even the locomotor activity of *An*. *aquasalis* [39, 48]. Therefore, IVM MDA has strong potential to be a novel tool for malaria transmission control. To our knowledge, this is the first study to evaluate mIVM effects on the *P*. *vivax* oocyst infection and intensity in *Anopheles*. This is also the first study to asses IVM effects against *P*. *vivax* asexual blood-stage development.

Herein, it was demonstrated that IVM reduces the oocyst infection and intensity of *P*. *vivax*. When *An*. *aquasalis* were fed with different concentrations of pIVM, a reduction of oocyst infection rate and infection intensity at the 10, 20 and 40 ng/ml concentrations (Fig 2). These ivermectin concentrations were selected based on the human pharmacokinetic curve for IVM which corresponds to approximately the concentration found in human blood between 4 to 60 hours post ingestion of IVM at 200 μg/kg [33, 55]. A recent report demonstrates that *An*. *darlingi* had modest sporontocidal pIVM results wherein oocyst prevalence was significantly reduced by 22.6% at the lethal concentration that kills 50% (LC_50_) (43.2 ng/ml) and 17.1% at the LC_25_ (27.8 ng/ml) but not significantly by 11.3% at the LC_5_ (14.8 ng/ml). Furthermore, there were no reductions in oocyst intensity in *An*. *darlingi* at any pIVM concentration tested [43]. Other reports also show the effect of pIVM on *P*. *vivax* oocyst prevalence and intensity in Asian vectors *Anopheles dirus* and *Anopheles minimus* wherein sporontocidal effect was far more impactful [38].

Interestingly, when *An*. *aquasalis* was fed *P*. *vivax* with mIVM, a reduction on infection rate and intensity was observed with plasma collected at 4 hours, 1 and 5 days after drug intake by the healthy volunteers. A far more potent sporontocidal effect was observed in *An*. *aquasalis* ingesting mIVM concentrations from day 1 (5.55 ng/ml) reduced oocyst prevalence by 51.5% compared to pIVM at 5 ng/ml by 12.6%. A similar effect was observed in *An*. *darlingi* ingesting mIVM concentrations from day 1 (5.69 ng/ml) reduced oocyst prevalence by 73.9% compared to pIVM at 14.8 ng/ml reduced oocyst prevalence by 11.3% [43]. Here we demonstrate that metabolized ivermectin has a more potent sporontocidal effect compared to ivermectin compound (Fig 4), which suggests that ivermectin metabolites may enhance the sporontocidal effect. Little is known about ivermectin metabolite production in humans, one small trial (n = 4) indicated that mean peak plasma concentration of metabolites was 2.5 fold greater than that of the parent compound and the effective half-lives of the metabolites were approximately 2.9 days while the parent compound half-life was 11.8 hours [59]. Future studies should be designed to elucidate ivermectin metabolite production in orally-treated volunteers and their impact on mosquito survivorship and *Plasmodium* sporogony. Interestingly, oocyst prevalence and intensity was more intensely impacted with mIVM plasma from 1 day in *An*. *darlingi* compared to *An*. *aquasalis* (Fig 3) but no significant reduction occurred when plasma from day 5 was fed to *An*. *darlingi* which suggests a shorter duration of but stronger sporontocidal effect compared to *An*. *aquasalis*.

Mosquito mortality effect was observed at day 7 post-blood feed with mIVM plasma from 4 hours and 1 day in the two-species studied. This IVM effect on mosquito survival is similar to our previous findings in non-infected *An*. *aquasalis* which also used metabolized ivermectin showing the higher impact on the survival and reproductive fitness [39] and with other studies in different anopheline species infected and uninfected with *Plasmodium* [9, 50, 60, 61]. In a single dose of 200 mcg/kg showed an increase in mosquito mortality in *An*. *aquasalis* when fed on mIVM at 1 day (5.55 ng/ml) to 5 days (1.58 ng/ml) the drug intake, but there was no longer effect when fed plasma collected from days 10 or 14.

The reduction in *An*. *aquasalis* and *An*. *darlingi* oocyst infection and intensity when mosquitoes were blood fed mIVM 4 days after infection expands the window that a blood meal containing IVM has an effect on *P*. *vivax* mosquito infection. Moreover, IVM was able to impair parasite development even when it was given to the mosquitoes after the midgut epithelium invasion by parasite ookinete suggesting that IVM has direct effects on already established and developing oocysts. Interestingly, these results were different from Kobylinski et al. [45], wherein no sporontocidal effect was observed on early-stage oocyst development when pIVM was fed 3 days after *P*. *falciparum* infection in *An*. *gambiae*.

This study also evaluates the *in vivo* exposure of *P*. *vivax* to IVM, CQ, and PQ in the human and its subsequent development in *An*. *aquasalis*. Mosquito oocyst infection and intensity were significantly reduced when mosquitoes were fed blood from patients treated with IVM+CQ, PQ+CQ or IVM+PQ+CQ but not CQ alone (Fig 6). Importantly, this is the first study to show that primaquine has a sporontocidal effect on *P*. *vivax* infection in the mosquito. However, the reduction on infection rate and intensity were not augmented on IVM +PQ+ CQ treated blood in relation to IVM+CQ only, suggesting that PQ does not have an additive or synergistic effect beyond IVM.

The mosquitoes were exposed in parallel to *P*. *vivax* with unprocessed and reconstituted blood samples. Unprocessed blood samples allowed for *in vivo* exposure of *P*. *vivax* to the drugs in the human compared to reconstituted blood samples which investigated the impact of metabolized drugs on *P*. *vivax*. The reduction in oocyst infection and intensity found in IVM+CQ and IVM+PQ+CQ treatment groups was similar in mosquitoes fed with the unprocessed and reconstituted blood (Fig 6). These data indicate that the ivermectin transmission blocking effect occurs in the mosquito midgut and not in human blood. Similar results were observed in the PQ+CQ and IVM+PQ+CQ groups, suggesting that primaquine effect on mosquito infection also occurs in the midgut. Since these assays were performed at only one-time point after drug intake (4 hours), we could not discard a possible delayed *in vivo* effect of primaquine or ivermectin on *P*. *vivax* asexual stages and gametocytes in the patient.

It is important to note that all patients who received the different regimens treatment were also supplied with CQ at the same time, following the guidelines of the Brazilian Health Ministry, which recommend all the patients ethically have to receive the CQ treatment at the same time that they are diagnosed. As expected, the mosquitoes fed a blood meal containing only CQ did not show a decrease in the oocyst infection prevalence of *An*. *aquasalis* with *P*. *vivax*, which is in accordance with other studies, where CQ did not affect the oocyst prevalence of *Plasmodium berghei* in *An*. *gambiae* [62]. However, this is the first report to demonstrate that oocyst intensity was reduced in mosquitoes fed a blood meal containing CQ. A reduction in oocyst intensity by CQ could be due to some direct action on *P*. *vivax*, or its immunosuppressive potential [63] may interfere with successful parasite midgut invasion leading to fewer oocysts.

Our results showed the highest mortality rate reduction of infection rate and intensity of *An*. *aquasalis* and *An*. *darlingi*, two important vectors of South American on plasma 4 hours and considering that in the human pharmacokinetic curve of the IVM the mean peak plasma concentrations is (46.6 ± 21.9ng/ml) at approximately 4 hours after dosing, with a IVM half-life from about 12 to 56 hours [33]. Similar peaks have been found in Primaquine and Chloroquine [64]. We can suggest the use of the IVM as a potent way to administration in combination with the other antimalaria drugs and mainly during the first hours after being detected the infection in the patient, which would have a higher impact in the Malaria elimination and eradication programs, specially, in endemics areas like Amazonas Region, which, have high incidence of Malaria cases by *P*. *vivax*.

This is the first study to assess the effect of ivermectin against asexual *P*. *vivax*. Two previous studies demonstrated an inhibition of pIVM on *P*. *falciparum* asexual stage development but with IC_50_s in the 1–10 μg/ml range [51, 65]. No effect of pIVM (3.9–1000 ng/ml) was observed in the current study against asexual blood-stage *P*. *vivax*, but this may have been due to using too low concentration (Fig 7). On the other hand, when asexual *P*. *vivax* was incubated with 4 different dilutions (4.28, 8.56, 17.12 and 34.24 ng/ml) of plasma obtained from healthy volunteers 4 hours after IVM administration, there was a significant decrease in *P*. *vivax* maturation in relation to the drug free control and incubations with plasma from healthy volunteers collected before IVM administration. It is important to highlight in the present study that when the asexual *P*. *vivax* was incubated with the pIVM (3.9 – 1000ng/ml) the development was not affected, however, a considerable reduction in blood-stage development was observed when the asexual stages were incubated with mIVM (4.28–34.24 ng/ml). This discrepancy might be a result of IVM metabolites conferring the parasite maturation inhibition effect. It is important to note that mIVM concentrations that showed blood-stage inhibition were achieved following oral administration with a standard dose of ivermectin (200 μg/kg). Unfortunately, data collected in this study could not be used to elucidate the ivermectin mechanism of action against asexual *P*. *vivax*. Further studies are warranted to evaluate the safety and efficacy of ivermectin as an adjunct during *P*. *vivax* antimalarial therapy.

We also have assayed the *P*. *vivax* sensitivity to pCQ on the same isolates used to examine the asexual maturation inhibition with IVM. These results showed pCQ IC_50_ values ranging from 1.96 ng/mL to 7.53ng/mL, similar to other reports [66–69], which demonstrate the chloroquine effect on *P*. *vivax*. Our findings also confirm the effect of chloroquine in terms of its pharmacodynamics against *P*. *vivax*.

In conclusion, our study shows for the first time the effect of mIVM on the oocyst infection and intensity of *P*. *vivax* in the South American malaria vectors *An*. *aquasalis* and *An*. *darlingi*. In both vectors it appears that mIVM has a stronger sporontocidal effect compared to pIVM, this suggests that ivermectin metabolites have sporontocidal effect. We report for the first time, the effect of IVM on *ex vivo* cultures of *P*. *vivax* and demonstrate that mIVM can inhibit *P*. *vivax* development. Moreover, it provides evidence that IVM may affect several parameters of Ross-MacDonald model [70], including parasite life cycle stages, placing it as a strong candidate for malaria transmission reduction.

## Supporting information

S1 Table Plasma ivermectin concentrations data. (DOCX)

S1 Dataset Study database. (XLSX)
