# Supplementary material for: Promising approach to reducing Malaria transmission by ivermectin: Sporontocidal effect against Plasmodium vivax in the South American vectors Anopheles aquasalis and Anopheles darlingi
Source: PLoS Negl Trop Dis. 2018 Feb 14;12(2):e0006221. doi: 10.1371/journal.pntd.0006221 (PMC5828505; doi:10.1371/journal.pntd.0006221)
Supplement: S1 Table — (DOCX) [file pntd.0006221.s001.docx]

| Sample Name | Analyte Area(cps) | IS Area(cps) | Dilution Factor | Calculated Concentration (ng/mL) | Analyte RT(min) |
| --- | --- | --- | --- | --- | --- |
| Volunteer 01-1 (1 – hr4 #1) | 8.52e+04 | 3.88e+05 | 1.0 | 81.6 | 2.01 |
| Volunteer 01-2 (1 – hr4 #2) | 9.55e+04 | 4.36e+05 | 1.0 | 81.6 | 2.02 |
| Volunteer 01-1 (1 – d1 #1) | 9.33e+03 | 4.07e+05 | 1.0 | 8.44 | 2.01 |
| Volunteer 01-2 (1 – d1 #2) | 8.91e+03 | 3.58e+05 | 1.0 | 9.18 | 2.01 |
| Volunteer 01-1 (1 – d5#1) | 2.24e+03 | 3.96e+05 | 1.0 | 1.99 | 2.02 |
| Volunteer 01-2 (1 – d5#2) | 1.78e+03 | 3.72e+05 | 1.0 | 1.67 | 2.01 |
| Volunteer 01-1 (1 – d10#1) | 0.00e+00 | 3.93e+05 | 1.0 | N/A | 0.00 |
| Volunteer 01-2 (1 – d10#2) | 0.00e+00 | 3.83e+05 | 1.0 | N/A | 0.00 |
| Volunteer 01-1 (1 – d14#1) | 0.00e+00 | 3.63e+05 | 1.0 | N/A | 0.00 |
| Volunteer 01-2 (1 – d14#2) | 0.00e+00 | 3.68e+05 | 1.0 | N/A | 0.00 |
| Volunteer 02-1 (2 – hr4#1) | 8.49e+04 | 3.69e+05 | 1.0 | 85.7 | 2.01 |
| Volunteer 02-2 (2 – hr4#2) | 7.53e+04 | 3.79e+05 | 1.0 | 73.9 | 2.01 |
| Volunteer 02-1 (2 – d1#1) | 8.28e+03 | 4.11e+05 | 1.0 | 7.40 | 2.01 |
| Volunteer 02-2 (2 – d1#2) | 8.22e+03 | 4.26e+05 | 1.0 | 7.08 | 2.02 |
| Volunteer 02-1 (2 – d2#1) | 4.48e+03 | 3.81e+05 | 1.0 | 4.27 | 2.01 |
| Volunteer 02-2 (2 – d2#2) | 5.10e+03 | 3.96e+05 | 1.0 | 4.69 | 2.01 |
| Volunteer 02-1 (2 – d5#1) | 2.09e+03 | 3.96e+05 | 1.0 | 1.86 | 2.01 |
| Volunteer 02-2 (2 – d5#2) | 2.22e+03 | 4.10e+05 | 1.0 | 1.91 | 2.01 |
| Volunteer 02-1 (2 – d10#1) | 0.00e+00 | 3.81e+05 | 1.0 | N/A | 0.00 |
| Volunteer 02-2 (2 – d10#2) | 0.00e+00 | 3.84e+05 | 1.0 | N/A | 0.00 |
| Volunteer 03-1 (3 – hr4#1) | 5.16e+04 | 3.83e+05 | 1.0 | 50.1 | 2.02 |
| Volunteer 03-2 (3 – hr4#2) | 5.29e+04 | 3.85e+05 | 1.0 | 51.1 | 2.01 |
| Volunteer 03-1 (3 – d1#1) | 1.05e+04 | 3.68e+05 | 1.0 | 10.6 | 2.01 |
| Volunteer 03-2 (3 – d1#2) | 1.18e+04 | 3.92e+05 | 1.0 | 11.1 | 2.01 |
| Volunteer 03-1 (3 – d2#1) | 5.67e+03 | 3.93e+05 | 1.0 | 5.27 | 2.01 |
| Volunteer 03-2 (3 – d2#2) | 6.12e+03 | 4.23e+05 | 1.0 | 5.28 | 2.01 |
| Volunteer 03-1 (3 – d5#1) | 3.12e+03 | 3.54e+05 | 1.0 | 3.17 | 2.01 |
| Volunteer 03-2 (3 – d5#2) | 3.27e+03 | 4.09e+05 | 1.0 | 2.87 | 2.01 |
| Volunteer 03-1 (3 – d10#1) | 0.00e+00 | 3.42e+05 | 1.0 | N/A | 0.00 |
| Volunteer 03-2 (3 – d10#2) | 0.00e+00 | 4.03e+05 | 1.0 | N/A | 0.00 |
| Volunteer 03-1 diluted 10 times (3 – d14#1) | 0.00e+00 | 3.93e+05 | 10.0 | N/A | 0.00 |
| Volunteer 03-2 diluted 10 times (3 – d14#2) | 0.00e+00 | 3.76e+05 | 10.0 | N/A | 0.00 |
| Volunteer 04-1 (4 – hr4#1) | 7.65e+04 | 3.76e+05 | 1.0 | 75.7 | 2.01 |
| Volunteer 04-2 (4 – hr4#2) | 7.09e+04 | 3.44e+05 | 1.0 | 76.8 | 2.01 |
| Volunteer 04-1 (4 – d1#1) | 1.09e+04 | 3.57e+05 | 1.0 | 11.2 | 2.01 |
| Volunteer 04-2 (4 – d1#2) | 1.09e+04 | 3.70e+05 | 1.0 | 10.8 | 2.02 |
| Volunteer 04-1 (4 – d5#1) | 2.75e+03 | 3.65e+05 | 1.0 | 2.69 | 2.02 |
| Volunteer 04-2 (4 – d5#2) | 3.23e+03 | 3.64e+05 | 1.0 | 3.20 | 2.01 |
| Volunteer 04-1 (4 – d10#1) | 3.04e+03 | 3.55e+05 | 1.0 | 3.07 | 2.01 |
| Volunteer 04-2 (4 – d10#2) | 2.20e+03 | 3.53e+05 | 1.0 | 2.21 | 2.02 |
| Volunteer 04-1 (4 – d14#1) | 1.71e+03 | 3.48e+05 | 1.0 | 1.72 | 2.01 |
| Volunteer 04-2 (4 – d14#2) | 2.28e+03 | 4.00e+05 | 1.0 | 2.01 | 2.02 |
| Volunteer 05-1 diluted 10 times (5 – hr4#1) | 1.06e+04 | 3.47e+05 | 10.0 | 113. | 2.01 |
| Volunteer 05-2 diluted 10 times (5 – hr4#2) | 1.08e+04 | 3.64e+05 | 10.0 | 110. | 2.02 |
| Volunteer 05-1 (5 – d1#1) | 8.32e+03 | 3.61e+05 | 1.0 | 8.49 | 2.02 |
| Volunteer 05-2 (5 – d1#2) | 9.35e+03 | 3.76e+05 | 1.0 | 9.16 | 2.01 |
| Volunteer 05-1 (5 – d5#1) | 4.68e+03 | 3.81e+05 | 1.0 | 4.47 | 2.01 |
| Volunteer 05-2 (5 – d5#2) | 4.66e+03 | 3.95e+05 | 1.0 | 4.28 | 2.01 |
| Volunteer 05-1 (5 – d10#1) | 2.34e+03 | 3.73e+05 | 1.0 | 2.22 | 2.01 |
| Volunteer 05-2 (5 – d10#2) | 2.65e+03 | 3.92e+05 | 1.0 | 2.41 | 2.01 |
| Volunteer 05-1 diluted 10 times (5 – d14#1) | 0.00e+00 | 3.67e+05 | 10.0 | N/A | 0.00 |
| Volunteer 05-2 diluted 10 times (5 – d14#2) | 0.00e+00 | 4.41e+05 | 10.0 | N/A | 0.00 |
| Patient 01-1 | 0.00e+00 | 4.12e+05 | 1.0 | N/A | 0.00 |
| Patient 01-2 | 0.00e+00 | 3.73e+05 | 1.0 | N/A | 0.00 |
| Patient 02-1 | 5.19e+04 | 4.02e+05 | 1.0 | 48.0 | 2.01 |
| Patient 02-2 | 5.07e+04 | 4.17e+05 | 1.0 | 45.2 | 2.01 |
| Patient 03-1 | 2.12e+03 | 3.55e+05 | 1.0 | 2.11 | 2.02 |
| Patient 03-2 | 2.11e+03 | 3.57e+05 | 1.0 | 2.09 | 2.01 |
| Patient 04-1 | 2.24e+04 | 4.05e+05 | 1.0 | 20.4 | 2.02 |
| Patient 04-2 | 2.05e+04 | 3.81e+05 | 1.0 | 20.0 | 2.02 |
| Patient 05-1 | 7.94e+03 | 3.63e+05 | 1.0 | 8.04 | 2.02 |
| Patient 05-2 | 7.97e+03 | 4.30e+05 | 1.0 | 6.79 | 2.01 |
| Patient 06-1 | 2.86e+03 | 3.89e+05 | 1.0 | 2.63 | 2.02 |
| Patient 06-2 | 3.31e+03 | 3.84e+05 | 1.0 | 3.10 | 2.01 |

### Peak Review

IVM-1


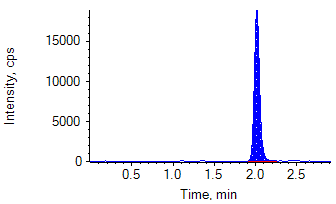


KJ01-1

D2 IVM-1


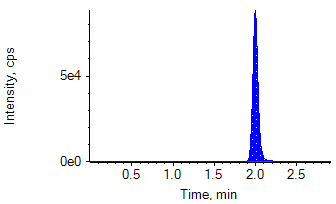


KJ01-1

IVM-1


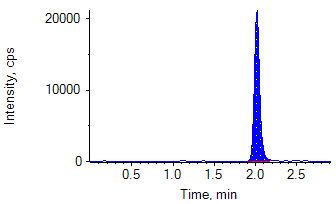


KJ01-2

D2 IVM-1


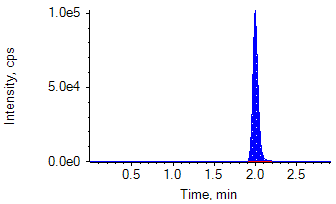


KJ01-2

IVM-1


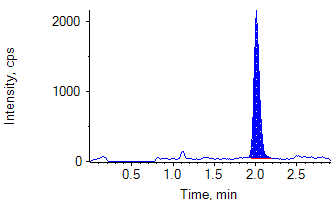


KJ02-1

D2 IVM-1


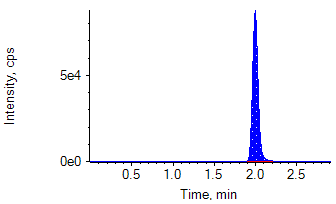


KJ02-1

IVM-1


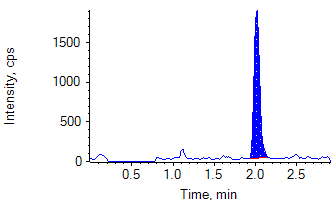


KJ02-2

D2 IVM-1


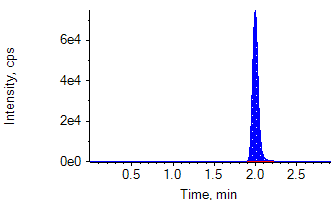


KJ02-2

IVM-1


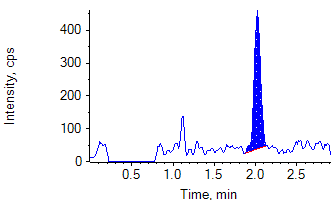


KJ03-1

D2 IVM-1


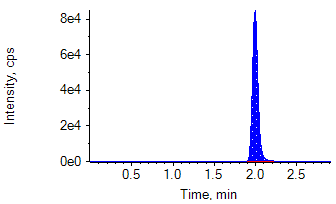


KJ03-1

IVM-1


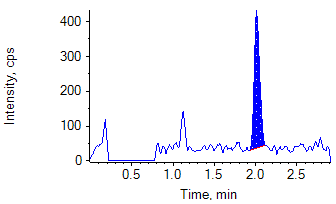


KJ03-2

D2 IVM-1


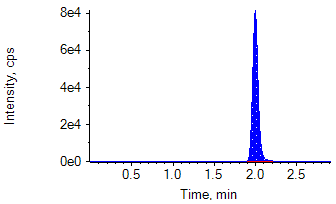


KJ03-2

IVM-1


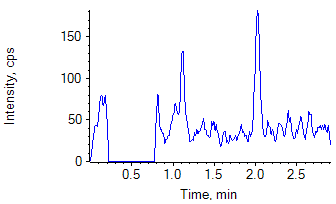


KJ04-1

D2 IVM-1


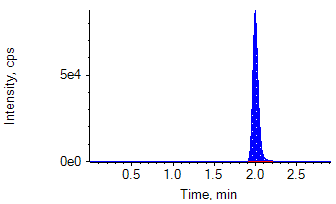


KJ04-1

IVM-1


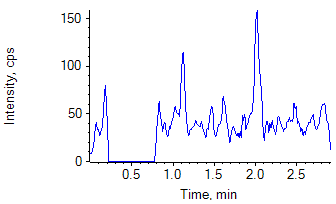


KJ04-2

D2 IVM-1


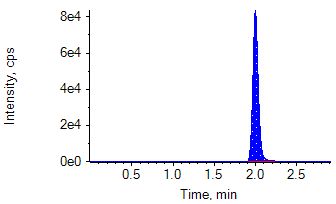


KJ04-2

IVM-1


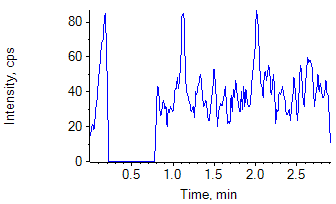


KJ05-1

D2 IVM-1


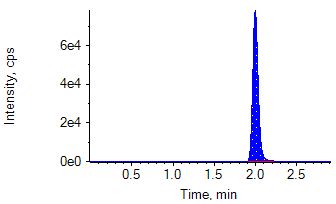


KJ05-1

IVM-1


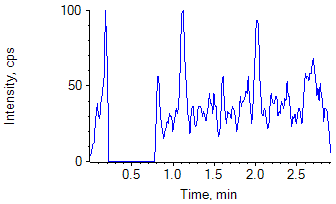


KJ05-2

D2 IVM-1


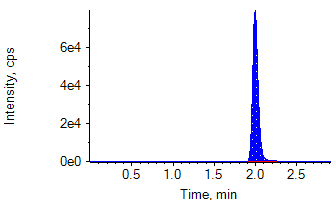


KJ05-2

IVM-1


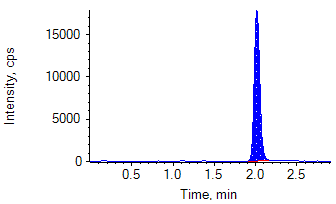


KJ06-1

D2 IVM-1


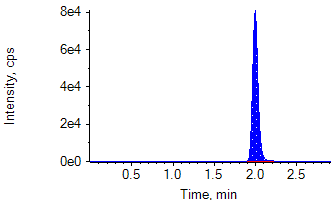


KJ06-1

IVM-1


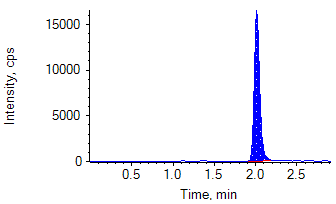


KJ06-2

D2 IVM-1


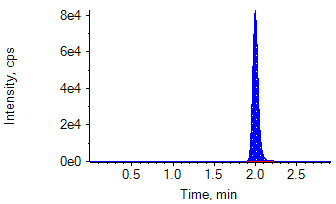


KJ06-2

IVM-1


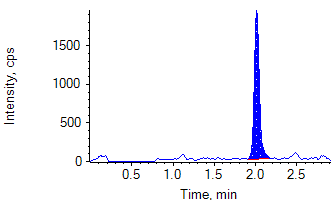


KJ07-1

D2 IVM-1


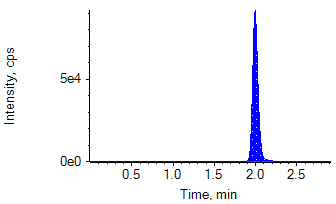


KJ07-1

IVM-1


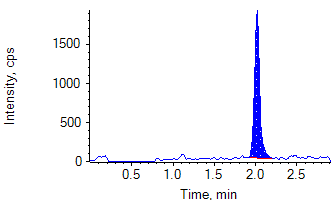


KJ07-2

D2 IVM-1


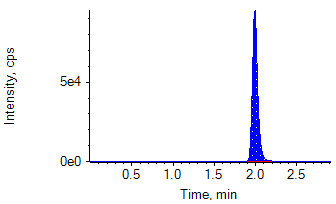


KJ07-2

IVM-1


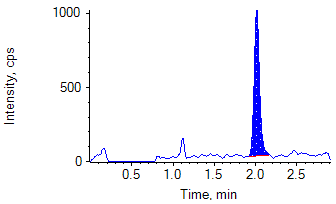


KJ08-1

D2 IVM-1


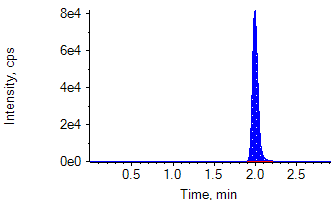


KJ08-1

IVM-1


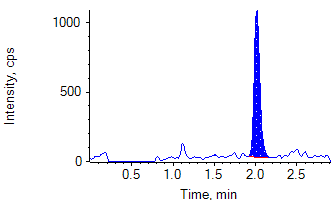


KJ08-2

D2 IVM-1


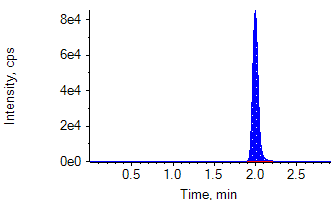


KJ08-2

IVM-1


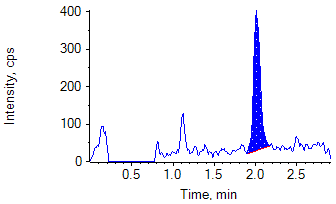


KJ09-1

D2 IVM-1


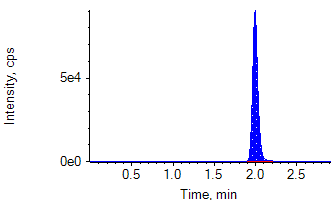


KJ09-1

IVM-1


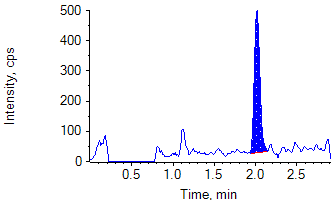


KJ09-2

D2 IVM-1


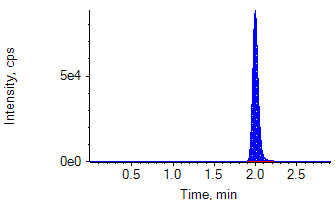


KJ09-2

IVM-1


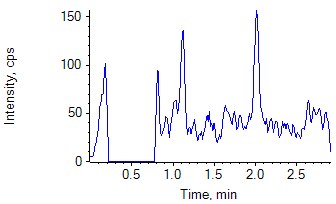


KJ10-1

D2 IVM-1


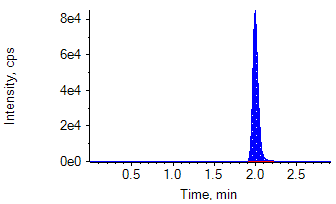


KJ10-1

IVM-1


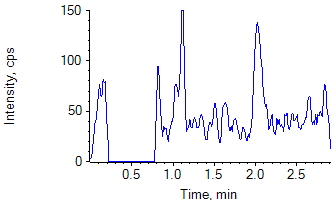


KJ10-2

D2 IVM-1


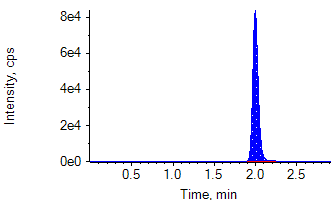


KJ10-2

IVM-1


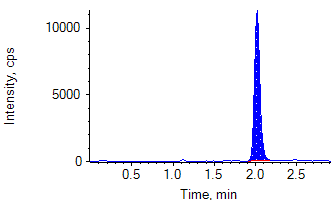


KJ11-1

D2 IVM-1


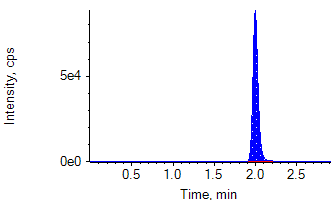


KJ11-1

IVM-1


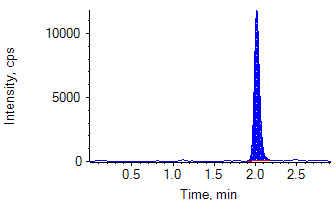


KJ11-2

D2 IVM-1


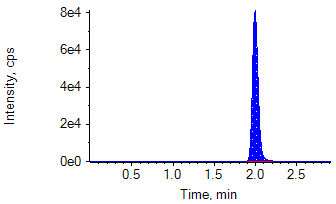


KJ11-2

IVM-1


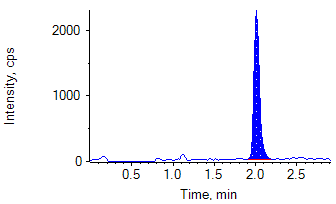


KJ12-1

D2 IVM-1


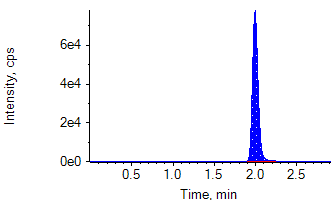


KJ12-1

IVM-1


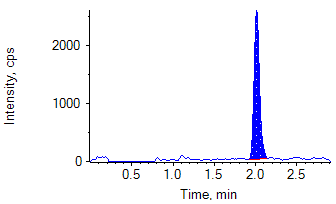


KJ12-2

D2 IVM-1


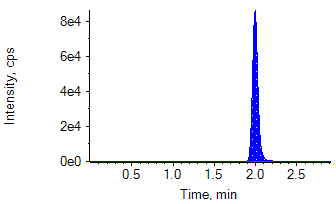


KJ12-2

IVM-1


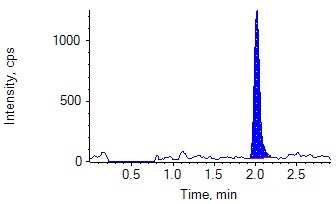


KJ13-1

D2 IVM-1


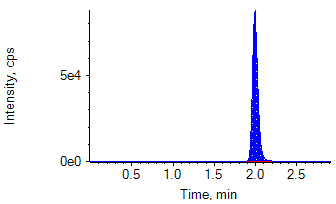


KJ13-1

IVM-1


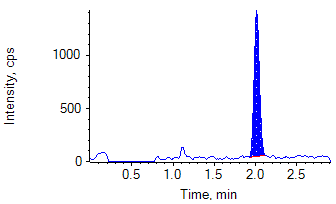


KJ13-2

D2 IVM-1


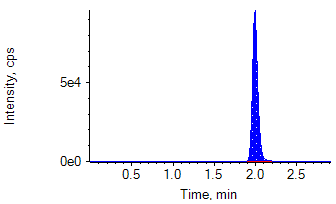


KJ13-2

IVM-1


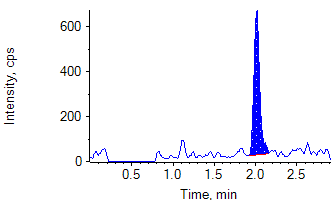


KJ14-1

D2 IVM-1


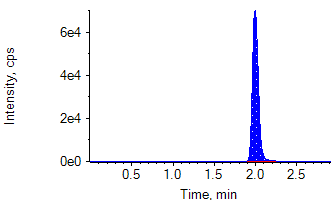


KJ14-1

IVM-1


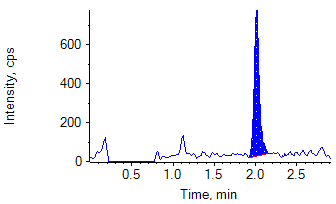


KJ14-2

D2 IVM-1


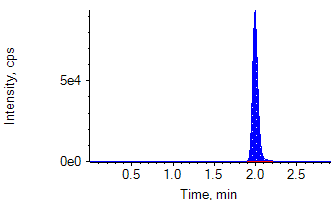


KJ14-2

IVM-1


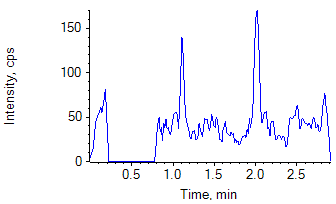


KJ15-1

D2 IVM-1


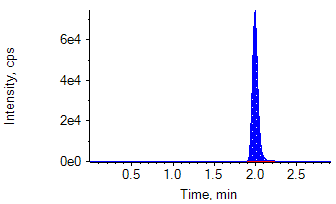


KJ15-1

IVM-1


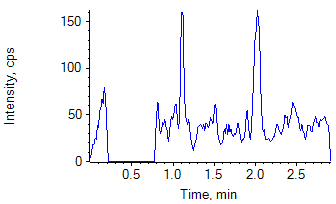


KJ15-2

D2 IVM-1


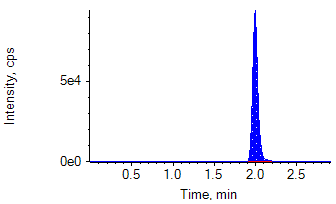


KJ15-2

IVM-1


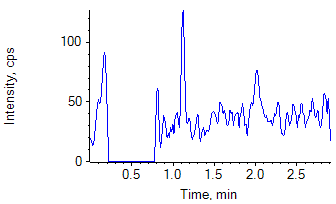


KJ16-1 diluted 10 times

D2 IVM-1


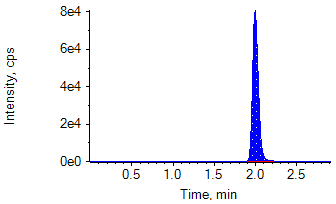


KJ16-1 diluted 10 times

IVM-1


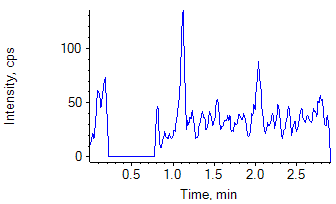


KJ16-2 diluted 10 times

D2 IVM-1


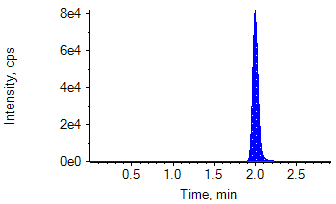


KJ16-2 diluted 10 times

IVM-1


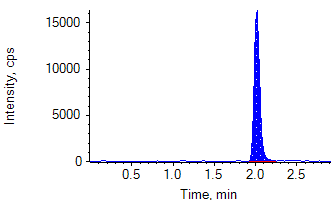


KJ17-1

D2 IVM-1


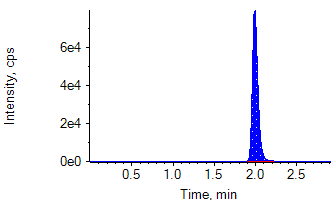


KJ17-1

IVM-1


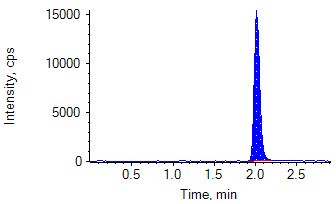


KJ17-2

D2 IVM-1


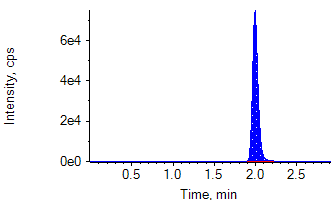


KJ17-2

IVM-1


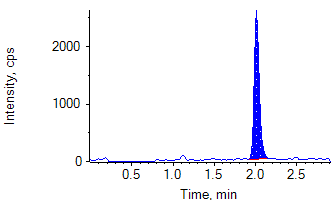


KJ18-1

D2 IVM-1


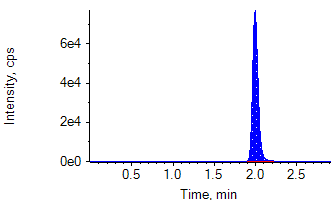


KJ18-1

IVM-1


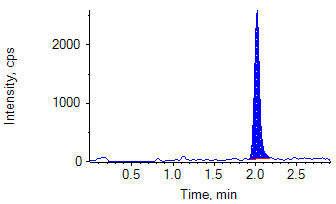


KJ18-2

D2 IVM-1


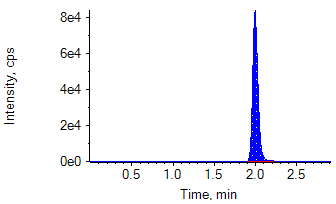


KJ18-2

IVM-1


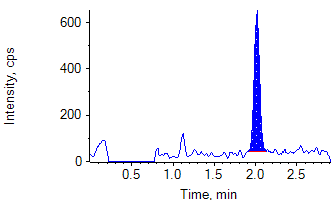


KJ19-1

D2 IVM-1


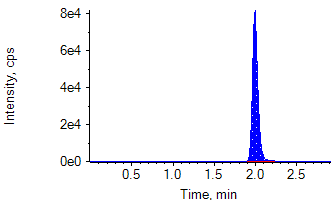


KJ19-1

IVM-1


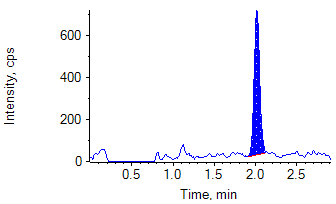


KJ19-2

D2 IVM-1


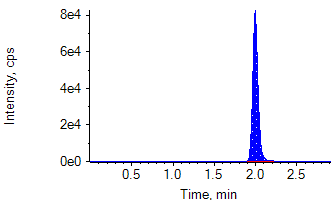


KJ19-2

IVM-1


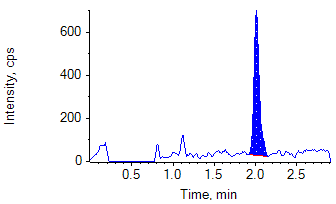


KJ20-1

D2 IVM-1


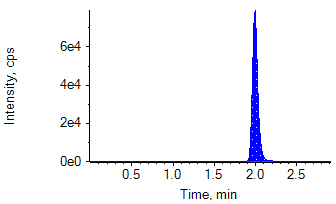


KJ20-1

IVM-1


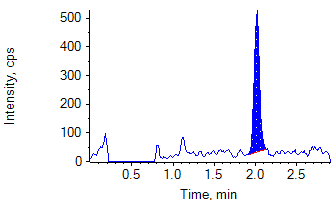


KJ20-2

D2 IVM-1


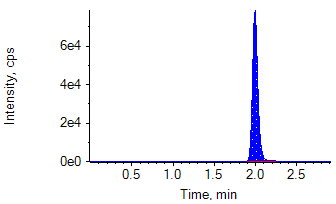


KJ20-2

IVM-1


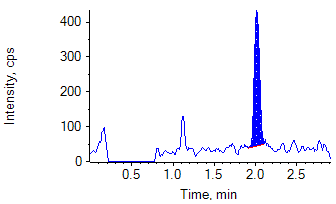


KJ21-1

D2 IVM-1


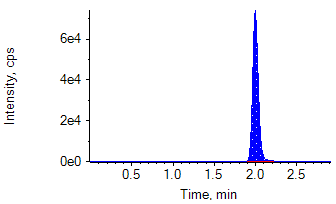


KJ21-1

IVM-1


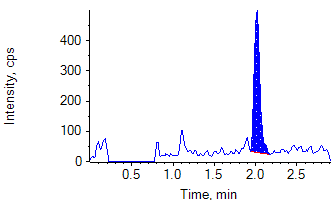


KJ21-2

D2 IVM-1


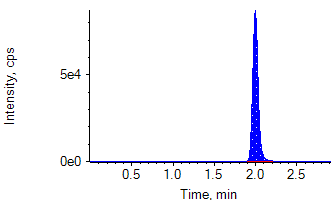


KJ21-2

IVM-1


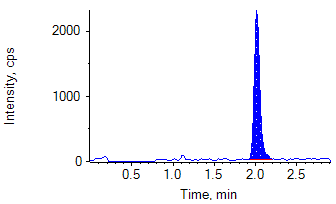


KJ22-1 diluted 10 times

D2 IVM-1


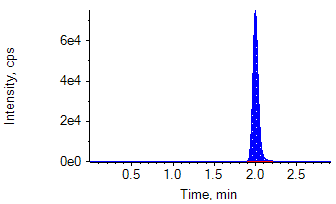


KJ22-1 diluted 10 times

IVM-1


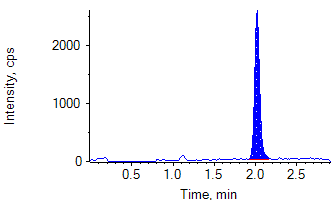


KJ22-2 diluted 10 times

D2 IVM-1


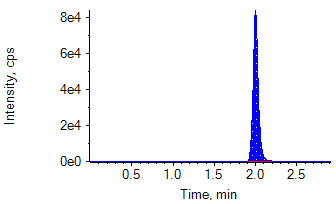


KJ22-2 diluted 10 times

IVM-1


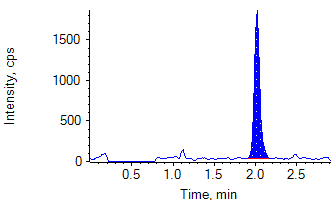


KJ23-1

D2 IVM-1


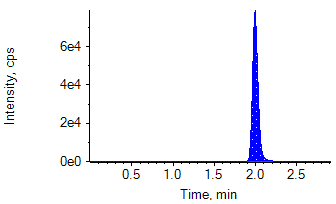


KJ23-1

IVM-1


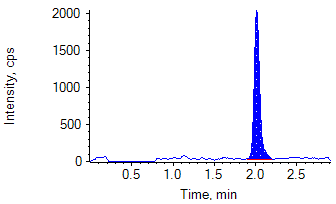


KJ23-2

D2 IVM-1


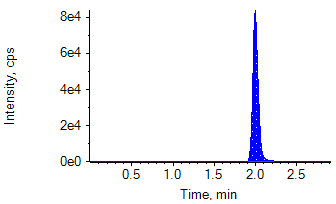


KJ23-2

IVM-1


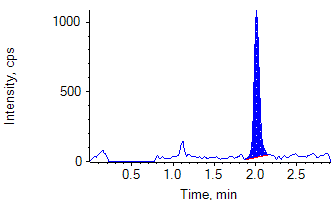


KJ24-1

D2 IVM-1


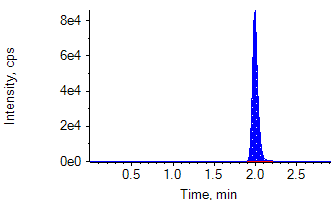


KJ24-1

IVM-1


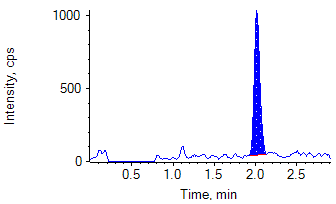


KJ24-2

D2 IVM-1


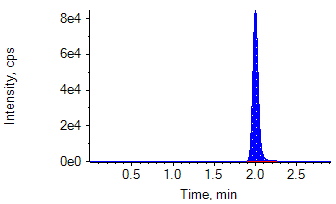


KJ24-2

IVM-1


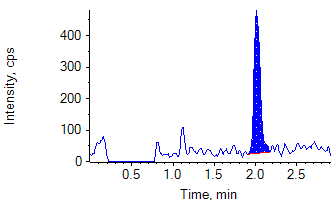


KJ25-1

D2 IVM-1


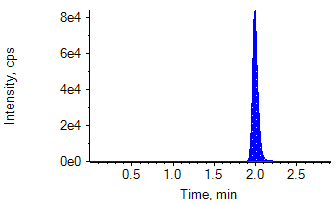


KJ25-1

IVM-1


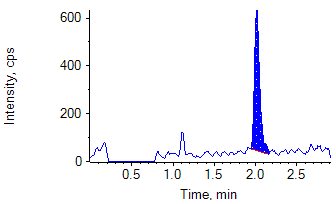


KJ25-2

D2 IVM-1


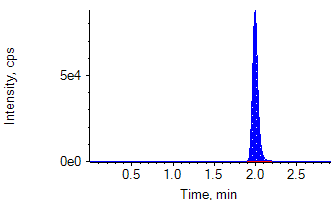


KJ25-2

IVM-1

KJ26-1 diluted 10 times

D2 IVM-1

KJ26-1 diluted 10 times

IVM-1

KJ26-2 diluted 10 times

D2 IVM-1

KJ26-2 diluted 10 times

IVM-1

KJ27-1

D2 IVM-1

KJ27-1

IVM-1

KJ27-2

D2 IVM-1

KJ27-2

IVM-1

KJ28-1

D2 IVM-1

KJ28-1

IVM-1

KJ28-2

D2 IVM-1

KJ28-2

IVM-1

KJ29-1

D2 IVM-1

KJ29-1

IVM-1

KJ29-2

D2 IVM-1

KJ29-2

IVM-1

KJ30-1

D2 IVM-1

KJ30-1

IVM-1

KJ30-2

D2 IVM-1

KJ30-2

IVM-1

KJ31-1

D2 IVM-1

KJ31-1

IVM-1

KJ31-2

D2 IVM-1

KJ31-2

IVM-1

KJ32-1

D2 IVM-1

KJ32-1

IVM-1

KJ32-2

D2 IVM-1

KJ32-2
